# Genome-wide characterization and identification of cyclophilin genes associated with leaf rust resistance in bread wheat (*Triticum aestivum* L.)

**DOI:** 10.3389/fgene.2022.972474

**Published:** 2022-09-30

**Authors:** Sandhya Tyagi, Shailendra Kumar Jha, Anuj Kumar, Gautam Saripalli, Ramesh Bhurta, Deepak T. Hurali, Lekshmy Sathee, Niharika Mallick, Reyazul Rouf Mir, Viswanathan Chinnusamy, Vinod

**Affiliations:** ^1^ Division of Plant Physiology, ICAR-Indian Agricultural Research Institute, New Delhi, India; ^2^ Division of Genetics, ICAR-Indian Agricultural Research Institute, New Delhi, India; ^3^ Centre for Agricultural Bioinformatics (CABin), Indian Agricultural Statistics Research Institute, New Delhi, India; ^4^ Department of Plant Science and Landscape Architecture, University of Maryland, College Park, MD, United States; ^5^ Division of Genetics and Plant Breeding, Faculty of Agriculture (FoA), Wadura Campus, Srinagar, India

**Keywords:** bread wheat, genome-wide identification, cyclophilin, leaf rust resistance, reactive oxygen species

## Abstract

Cyclophilins (CYPs) are a group of highly conserved proteins involved in host-pathogen interactions in diverse plant species. However, the role of CYPs during disease resistance in wheat remains largely elusive. In the present study, the systematic genome-wide survey revealed a set of 81 *TaCYP* genes from three subfamilies (GI, GII, and GIII) distributed on all 21 wheat chromosomes. The gene structures of *TaCY*P members were found to be highly variable, with 1–14 exons/introns and 15 conserved motifs. A network of miRNA targets with *TaCYPs* demonstrated that *TaCYPs* were targeted by multiple miRNAs and vice versa. Expression profiling was done in leaf rust susceptible Chinese spring (CS) and the CS-*Ae. Umbellulata* derived resistant IL “Transfer (TR). Three homoeologous *TaCYP* genes (*TaCYP*24, *TaCYP*31, and *TaCYP36*) showed high expression and three homoeologous *TaCYP* genes (*TaCYP44*, *TaCYP49*, and *TaCYP54*) showed low expression in TR relative to Chinese Spring. Most of the other TaCYPs showed comparable expression changes (down- or upregulation) in both contrasting TR and CS. Expression of 16 *TaCYP*s showed significant association (*p* < 0.05) with superoxide radical and hydrogen peroxide abundance, suggesting the role of *TaCYPs* in downstream signaling processes during wheat-leaf rust interaction. The differentially expressing *TaCYP*s may be potential targets for future validation using transgenic (overexpression, RNAi or CRISPR-CAS) approaches and for the development of leaf rust-resistant wheat genotypes.

## 1 Introduction

Bread wheat (*Triticum aestivum* L.) is considered as one of the most important cereal crops in the world. Various biotic and abiotic stresses severely hamper the production and productivity of the wheat crop. Among the biotic stresses, rusts constitute the most critical biotic stress. Out of three rusts affecting the wheat crop, leaf rust caused by *Puccinia triticina* L. is the most prevalent in almost all wheat-growing regions. Approximately 50% yield reduction has been reported when conditions are favourable for leaf rust infection (Huerta-Espino et al., 2011). The loss caused by leaf rust can be prevented by deploying resistant wheat cultivars possessing leaf rust resistance genes (Qiu et al., 2020). To date, ∼82 genes have been designated for leaf rust resistance in wheat (Mcintosh et al., 2014, 2017; Qiu et al., 2020; Kirti et al., 2020; Bariana et al., 2022), of which seven *Lr* genes have also been cloned, including seedling resistance (SR) genes such as *Lr1* (Cloutier et al., 2007)*, Lr10* (Feuillet et al., 2003)*, Lr21* (Huang et al., 2003)*, Lr22a* (Thind et al., 2017) and *Lr42* (Lin et al., 2022) and adult plant resistance genes (APRs) such as *Lr34* (Krattinger et al., 2019) and *Lr67* (Moore et al., 2015). Cyclophilins (CYPs) are a group of highly conserved proteins crucial in pathogenesis (A. Singh et al., 2017). The CYPs, along with FKBPs (FK506-binding proteins) (Harding et al., 1989) and the parvulins family (Gething, 1997) proteins, are members of the immunophilins group that have peptidylprolyl *cis*-trans activity (PPIase). In plants, the differential gene expression of CYPs has been observed in response to biotic stresses such as viral and fungal infection (Pandian et al., 2020; Olejnik et al., 2021) and abiotic stresses like drought, salinity, and temperature (Sharma and Taganna, 2020; Godoy et al., 2000; Marivet et al., 1992; Romano et al., 2004). Additionally, hormones such as salicylic acid (Marivet et al., 1992), jasmonic acid, methyl jasmonate (Wasternack and Strnad, 2016; Yan et al., 2016), abscisic acid (Godoy et al., 2000), and auxin (Bari & Jones, 2009), which are also known to be involved in signaling pathways during plant–pathogen interactions, have been reported to be involved in the regulation of CYP genes. For instance, in soybean, the expression of the *CYP* gene *CYP82A3* was found to be regulated by MeJA, which was also induced by different fungal infections (Yan et al., 2016).

Furthermore, the role of *CYP* gene family is well known in signaling pathways during plant–pathogen interactions, including *M. oryzae* (Wilson and Talbot, 2009), Phytophthora (Gan et al., 2009), and *Leptosphaeria maculans* (K. Singh et al., 2014), and during the *Arabidopsis-P. syringeae* interaction (Coaker et al., 2005). In Arabidopsis, the CYP gene was activates the bacterial effector *AvrRpt2,* leading to *RPS2*-mediated disease resistance against *Pseudomonas syringae* (Coaker et al., 2005).

The availability of complete genome sequencing data in public databases has paved the way for systematically identifying and annotating ∼16,000 *CYP* gene sequences in plant species (Gan et al., 2009; Pemberton & Kay, 2005; H. Singh et al., 2019; K. Singh et al., 2014). The *CYP* gene family has been characterized in *Arabidopsis thaliana, Oryza sativa, Glycine max, Zea mays, Solanum lycopersicum,* and *Gossypium hirsutum* (Gasser et al., 1990; Romano et al., 2004; Ahn et al., 2010; Mainali et al., 2014; Chen et al., 2019; Wang et al., 2020). In addition, several *CYP* genes involved in resistance against different biotic stresses have been reported in plants, including 1) *Nicotiana benthamiana*: overexpression of *GmCYP82A3* provides resistance to the black shank (*Phytophthora parasite*) and gray mold (*Botrytis cinerea*), 2) *Capsicum annum*: *CaCYP1* showed involvement in the hypersensitive response (HR) once plants were infected with *Xanthomonas axonoposis, and 3*) *Arabidopsis: AtCYP76C2* associated with hypersensitive cell death during infection with *Pseudomonas syringae.* Additionally, in wheat, a CYP member encoding for *CYP709C3v2* was found to be upregulated in the resistant genotype during *Fusarium* head blight infection caused by *Fusarium graminearum*, thereby indicating the role of CYP gene members during biotic stress tolerance in wheat.

The present work was planned to identify and characterize the *CYP* gene family in wheat during wheat-leaf rust interactions. Detailed *in silico* analysis was also conducted at the protein level, and essential motifs were identified that might be involved during resistance against leaf rust in wheat. The correlation of qRT-PCR expression data with reactive oxygen species (ROS) abundance, suggests a prominent role of *TaCYPs* in downstream signaling processes during wheat-leaf rust interaction.

## 2 Materials and methods

### 2.1 Genome-wide scanning of CYP genes in wheat

To identify the potential candidate CYP genes in the wheat genome, the protein sequences of CYP candidate genes from Arabidopsis, rice, and soybean were retrieved from TAIR (https://www.arabidopsis.org/index.jsp), The Rice Annotation Project database rap-db (https://rapdb.dna.affrc.go.jp), and PlantGDB database (http://www.plantgdb.org/) for *Glycine max,* respectively, were used as queries to find the homologs in wheat. Amino acid sequences of the previously reported *A. thaliana* cyclophilin-like peptidylprolyl *cis*-trans isomerase genes *AtCYP18-3* (Coaker et al., 2005) and *AtCYP19-1* (Pogorelko et al., 2014) were used as queries in a BLASTp algorithm to identify all the potential wheat *CYP* genes (*TaCYPs*) containing single or multiple domains. The BLASTp search was performed against the *T. aestivum* IWGSC (https://www.wheatgenome.org/) (protein) data, available on EnsemblPlants release 47 (https://plants.ensembl.org/index.html). All the protein sequences with an E-value below 1.0 and showing >85% similarity were retrieved. For the sequences with more than one transcripts, the primary transcript with the longest length was preferred as the emissary of genes (Hurali et al., 2021; Bhurta et al., 2022). The two databases, InterPro (Finn et al., 2017) and PROSITE (Sigrist et al., 2012), were used to identify the specific domains in all three recognized TaCYP proteins. The genomic sequences, DNA sequences, and coding domain sequences (CDSs) of all the identified *TaCYP* genes were downloaded from the EnsemblPlants release 47 (https://plants.ensembl.org/index.html) data set using the assigned Ensemble transcripts Ids.

### 2.2 Physical mapping of *TaCYP* genes on wheat chromosomes

All the identified *TaCYP* genes were physically mapped onto seven homoeologous chromosome groups using information available in public repositories, including IWGSC-URGI (https://wheat-urgi.versailles.inra.fr/) and EnsemblPlants release 47 (https://plants.ensembl.org/index.html).

### 2.3 Gene structure boundary prediction and conserved motif distribution

DNA sequences and coding domain sequences (CDSs) of all the identified *TaCYP* genes were used for gene structure analysis. A tool, Gene Structure Display Server (Hu et al., 2015), was used to predict the gene structure and exon–intron boundaries. Full-length protein sequences of predicted *TaCYP* genes were analyzed by MEME version 5.0.2 software (Bailey et al., 2009, 2015) to determine conserved motifs at the following parameters: 15 as the maximum number of motifs, with a restricted motif width of a minimum of 6 aa and maximum of 50 aa, while the other settings were default (Bhurta et al., 2022).

### 2.4 Phylogenetic analysis

Phylogenetic analysis was conducted to study the evolutionary relationship among the identified TaCYPs and the earlier CYPs reported in other plants. For this purpose, the CYP protein sequences of Arabidopsis (25 CYPs), rice (16 CYPs), and soybean (28 CYPs) were downloaded from TAIR (https://www.arabidopsis.org/), rap-db (https://rapdb.dna.affrc.go.jp/) and EnsemblPlants (https://plants.ensembl.org/index.html), respectively. Multiple sequence alignment (MSA) of amino acid sequences was performed using the ClustalW tool (http://ebi.ac.uk/Tools/msa/clustalW2). Evolutionary distances were measured using Molecular Evolutionary Genetics Analysis (MEGA 6.0). A phylogenetic tree was constructed using the neighbor-joining (NJ) algorithm with the substitution model, uniform rates, and pairwise deletion (Saitou and Nei, 1987), with bootstrap values for 1,000 iterations calculated and expressed as percentages (Felsenstein, 1985).

### 2.5 Identification of miRNAs and network analysis of miRNAs and *TaCYP* genes

The full-length genomic sequences of all the identified *TaCYPs* were mined as an input on the homology search-based psRNATarget server (Dai and Zhao, 2011) to determine the miRNAs targeting the *TaCYPs* with a selection of updated wheat miRNA libraries in the background. The potential miRNAs targeting the *TaCYPs* were identified with the following parameters embedded: maximum expectation: 2.0, length for complementarity scoring (HSP size): 19, penalty G:U pairs: 0.5, seed region: 2–13 nt, and extra weight in seed region: 1.5 (Kumar et al., 2019; Hurali et al., 2021; Bhurta et al., 2022). A desktop app of Cytoscape 3.5.1 (Shannon et al., 2003) was used to render the interaction network of miRNAs targeting *TaCYP* genes.

### 2.6 Physiochemical properties and subcellular localization of *TaCYP* genes

The amino acid sequences of all the selected *TaCYP* genes were screened for their physiochemical properties and subcellular localization. An automated ProtParam server available on the Expasy website (https://web.expasy.org/protparam/) (Gasteiger et al., 2005) was used to calculate the different physiochemical properties, including average residue weight (g/mol), charge, isoelectric point (IP), molecular weight (Mw), theoretical prediction of PI, instability index (II), aliphatic index (AI), grand average of hydropathicity (GRAVY) and stability. The subcellular localization of the identified TaCYP proteins was predicted by BUSCA (http://busca.biocomp.unibo.it) (Savojardo et al., 2018).

### 2.7 *In silico* tissue-specific expression analysis

Transcriptome expression data from expVIP (http://www.wheat-expression.com/) for two biotic stress treatments (stripe rust and powdery mildew) were used to compare the relative expression of the *TaCYPs*. A heatmap was generated using a wheat expression browser powered by expVIP (http://www.wheat-expression.com).

### 2.8 Plant materials

#### 2.8.1 Wheat genotypes

The leaf rust-susceptible wheat genotype “Chinese spring (CS)” and the CS-*Ae. Umbellulata* derived resistant IL “Transfer (Sears, 1956)” were used for differential gene expression analysis using qRT–PCR. TR wheat has a dominant seedling leaf rust resistance gene “*Lr9.*”

### 2.9 Pathogen

A single spore-derived inoculum of one of the most prevalent and virulent pathotypes, 77–5 (syn. 121R63–1) of *P. triticina* Eriks. was procured from Regional Station, Indian Institute of Wheat and Barley Research, Flowerdale, Shimla, India. The pathotype is avirulent against the seedling leaf rust resistance gene *Lr9* carried by TR and was used for inoculating the seedlings of the two wheat genetic stocks.

### 2.10 Inoculation at the seedling stage and collection of leaf samples

Wheat seedlings of CS (susceptible line) and TR (resistant line) were sown and raised in growth chambers under standardized, controlled conditions at the National Phytotron Facility, Indian Agricultural Research Institute (IARI), New Delhi (Prabhu et al., 2012). Seedlings were inoculated using the method described by (Dhariwal et al., 2011) and incubated for 48 h in a humid chamber (23 ± 2°C temperature). Standard conditions were restored for the seedlings after incubation. Random leaf samples were collected from seedlings of the CS and TR wheat lines 1) before inoculation, i.e., at 0 HBI (Hours Before Inoculation, uninoculated control), and 2) after seedling inoculation (HAI), i.e., at 24 HAI and 72 HAI with leaf rust pathotype 77–5.

The leaves of the two genotypes (CS and TR) were sampled at 24 HAI and 72 HAI to quantify superoxide radicals (SOR) and hydrogen peroxide (H_2_O_2_) localisation following the methodology described earlier (Qiao et al., 2015; and Bhurta et al., 2022). The spectrophotometric assay method described by (Chaitanya and Naithani 1994) was used to quantify SOR in fresh leaf tissue. The amount of NBT (nitroblue tetrazolium chloride) that was reduced by SOR was measured. Leaf samples (1 g) were ground in 0.2 M phosphate buffer (precooled, pH 7.2) and centrifuged at 10,000 g for 30 min at 4°C. The supernatant was collected, and an assay mixture was prepared (0.1 mM EDTA, 0.075 mM NBT, 13.33 mM L-methionine, 25 mM Na_2_CO_3_, 250 µl of supernatant in a final volume of 3 ml). The absorbance at 540 nm was measured using the assay mixture.

Leaf samples (1 g) were crushed in liquid nitrogen and homogenized in a 10 ml cooled acetone solution for H_2_O_2_ estimation. The homogenized solution was filtered using Whatman no. 1 filter paper, and the filtrate was mixed with a 5:4 ratio of ammonium solution (5 ml) and titanium reagent (5 ml). After centrifugation at 10,000 *g* for 10 min, the precipitated titanium-hydro peroxide complex was dissolved in 10 ml of 2 M H_2_SO_4_ and re-centrifuged. The supernatant was collected, and the spectroscopic absorbance was measured at 415 nm (Rao et al., 1997).

### 2.11 RNA isolation and cDNA preparation

Leaf tissue **(**50–100 mg) was collected from wheat CS (susceptible line) and TR (resistant line) seedlings for RNA isolation. Sigma’s TRI reagent kit was used to isolate RNA. RNase-free DNase I was used to treat total isolated RNA (Qiagen). According to the manufacturer’s instructions, a total of 2.0 μg isolated, purified RNA was used for cDNA synthesis (reverse transcription) using the Promega M-MuLV Reverse Transcriptase kit.

### 2.12 Primer design, quantitative real-time (qRT–PCR) and correlation of ROS with gene expression

The software Primer Express version 3.0 (Applied Biosystems, https://primer-express.software.informer.com/3.0/) was used to design primers for RT–PCR. The 81 *TaCYP* genes were grouped on the basis of their localisation on homoeologous chromosomes, length of amino acids, and the number of exon intron. A set of 25 primers were designed using the Primer Express program version 3.0 (Applied Biosystems) (length; 18–25 bases, GC content; 40%–60%, and Tm = 60 ± 1°C, product size; 70 and 150 bp) and used for qRT–PCR. The CFX96™ Real-time PCR Detection System (BioRad) performed qRT–PCR with Applied Biosystems SYBR Green PCR Master Mix. Each qRT–PCR was run (containing two biological replicates and three technical replicates each) with a total 20 μl reaction mixture, including 10 μl SYBR Premix Ex Taq, 2 μl cDNA, 0.8 μl forward primer, 0.8 μl reverse primer, and 6.4 μldd H_2_O in a 96-well optical plate, and was amplified according to the following thermal cycling conditions: 95°C for 10 s, followed by 40 cycles of 5 s at 95°C and 30 s at 60°C. The PCR product was heated from 65 to 95°C (0.5°C/5 s) to draw the melting curve, and the raw C^t^ values were obtained. The endogenous control gene of wheat (*TaAct2*), expressed constitutively, was used to normalize the data. Fold change values (2^−ΔΔCt^) for gene expression were calculated for both stress conditions vs. the control as explained by Thomas D Schmittgen (2008), as follows:

$$2^{-\Delta \Delta Ct}=[(C_{t}TaCYP-C_{t}TaAct)\;treated-(C_{t}TaCYP-C_{t}TaAct)\;control]$$

The transcript abundance for each gene was normalized to the internal control. Significance in the differential expression due to leaf rust infection (24 HAI and 72 HAI) was estimated through a paired *t* test using SPSS ver 16.0. Correlation of gene expression with ROS was estimated, and correlation values were depicted in the form of corrplot using the corrplot package (Friendly, 2002) available in R software.

### 2.13 Homology modeling and structure evaluation

Homology, also known as comparative modeling, is a powerful tool for predicting protein structure and function (Kumar et al., 2016). The 3D structure of TaCYP proteins was predicted using a homology modeling-based method, with solved structures of homologous proteins available in the Protein Data Bank (PDB) (https://www.rcsb.org/). Position-Specific Iterated BLAST (PSI-BLAST) (Altschul et al., 1997) was used against the PDB to identify suitable homologous template structures with a high score and lower e-value. Other criteria were previously described in (Gautam et al., 2019; Kumar et al., 2019; Mathpal, 2021). The TaCYP protein 3D structure was simulated using the Swiss-Model server (Arnold et al., 2006; Biasini et al., 2014). UCSF CHIMERA 1.10, a protein structure visualizer package (Pettersen et al., 2004), was used to render the predicted 3D structures in various 3D coordinates. To assess the expected structure models, a Ramachandran plot was calculated for each protein model by analyzing phi (Φ) and psi (Ψ) torsion angles and covalent bond quality using consensus algorithms from the PSVS (http://psvs-1.5-dev.nesg.org/) and SAVES servers (http://nihserver.mbi.ucla.edu/SAVES/).

## 3 Results

### 3.1 Identification of *TaCYP* gene members in wheat genome

Using the BLASTp search against the *T. aestivum* IWGSC (protein) data available on EnsemblPlants release 47 (https://plants.ensembl.org/index.html), a total of 81 *TaCYP* genes distributed on 21 bread wheat chromosomes were identified. According to their chromosomal positions, the 81 *TaCYP* genes were named *TaCYP1* to *TaCYP81*. All 81 identified sequences were further verified for their conserved domain using secondary databases, including InterPro and PROSITE (Table 1). Table 1 contains all 81 *TaCYP*s identified, including transcript ID, length of coding sequences (CDS) and amino acids (aa), chromosome location, coordinates, splice variants, and subcellular location. The size of the CDS of all 81 *TaCYP*s ranged from 465 bp (*TaCYP*75) to 2,550 bp (*TaCYP*50, and *TaCYP*55), and the corresponding aa length ranged from 154 aa (*TaCYP*75) to 849 aa (*TaCYP*50, and *TaCYP55*) (Table 1).

**TABLE 1:** Details of 81 *TaCYP* genes with their gene ID, length, chromosome location, coordinates, splice variants, and subcellular location.

S. No.	Gene	Ensemble ID	Splice variant	Splice selected	Strand	Coordinates	bp	aa	Exon	Coding exons	Genome location	Description (if known)
1	*TaCYP*1	TraesCS1A02G007500	1	TraesCS1A02G007500.1	F	4,045,713-4,048,684	1,146	245	7	7	Chromosome 1A: 4,045,713	
2	*TaCYP*2	TraesCS1B02G011100	1	TraesCS1B02G011100.1	F	5,157,094-5,160,358	1,350	245	7	7	Chromosome 1B: 5,157,094	Peptidyl-prolyl cis-trans isomerase
3	*TaCYP*3	TraesCS1D02G000800	2	TraesCS1D02G000800.2	R	216,466-219,484	1,322	245	7	7	Chromosome 1D: 216,466	Peptidyl-prolyl cis-trans isomerase
4	*TaCYP*4	TraesCS2A02G202300	2	TraesCS2A02G202300.1	R	176,671,654-176,677,679	1,640	459	13	12	Chromosome 2A: 176,671,654	No description
5	*TaCYP*5	TraesCS2A02G237700	2	TraesCS2A02G237700.1	R	313,770,244-313,778,042	2,404	643	14	13	Chromosome 2A: 313,770,244	No description
6	*TaCYP*6	TraesCS2B02G229400	1	TraesCS2B02G229400.1	R	224,649,128-224,655,926	2,857	459	12	12	Chromosome 2B: 224,649,128	No description
7	*TaCYP*7	TraesCS2B02G255000	1	TraesCS2B02G255000.1	R	288,470,184-288,504,135	1,282	233	8	7	Chromosome 2B: 288,470,184	Peptidyl-prolyl cis-trans isomerase
8	*TaCYP*8	TraesCS2B02G260600	1	TraesCS2B02G260600.1	R	329,472,516-329,486,857	2,453	635	14	13	Chromosome 2B: 329,472,516	No description
9	*TaCYP*9	TraesCS2D02G208600	4	TraesCS2D02G208600.1	F	163,013,438-163,020,415	2,863	424	12	12	Chromosome 2D: 163,013,438	No description
10	*TaCYP*10	TraesCS2D02G237600	1	TraesCS2D02G237600.1	F	242,325,816-242,332,524	1,105	233	8	7	Chromosome 2D: 242,325,816	Peptidyl-prolyl cis-trans isomerase
11	*TaCYP*11	TraesCS2D02G244700	1	TraesCS2D02G244700.1	R	276,931,184-276,939,618	2,437	636	14	13	Chromosome 2D: 276,931,184	No description
12	*TaCYP*12	TraesCS3A02G005900	1	TraesCS3A02G005900.1	R	7,200,517-7,201,780	1,148	295	2	2	Chromosome 3A: 7,200,517	Peptidyl-prolyl cis-trans isomerase
13	*TaCYP*13	TraesCS3A02G064900	1	TraesCS3A02G064900.1	F	38,316,582-38,317,799	1,218	405	1	1	Chromosome 3A: 38,316,582	No description
14	*TaCYP*14	TraesCS3A02G151100	1	TraesCS3A02G151100.1	F	140,003,720-140,014,025	1,207	240	6	6	Chromosome 3A: 140,003,720	Peptidyl-prolyl cis-trans isomerase
15	*TaCYP*15	TraesCS3A02G209000	1	TraesCS3A02G209000.1	R	370,243,187-370,250,719	1977	495	11	10	Chromosome 3A: 370,243,187	No description
16	*TaCYP*16	TraesCS3B02G008100	1	TraesCS3B02G008100.1	F	4,202,094-4,203,405	1,181	291	2	2	Chromosome 3B: 4,202,094	Peptidyl-prolyl cis-trans isomerase
17	*TaCYP*17	TraesCS3B02G178000	1	TraesCS3B02G178000.1	F	182,320,933-182,343,627	723	240	6	6	Chromosome 3B: 182,320,933	Peptidyl-prolyl cis-trans isomerase
18	*TaCYP*18	TraesCS3B02G239300	1	TraesCS3B02G239300.1	R	377,426,766-377,432,727	1868	500	11	10	Chromosome 3B: 377,426,766	No description
19	*TaCYP*19	TraesCS3D02G004600	1	TraesCS3D02G004600.1	F	1,845,275-1,846,487	1,108	295	2	2	Chromosome 3D: 1,845,275	No description
20	*TaCYP*20	TraesCS3D02G065600	1	TraesCS3D02G065600.1	F	28,965,940-28,967,059	1,005	334	3	3	Chromosome 3D: 28,965,940	No description
21	*TaCYP*21	TraesCS3D02G112800	1	TraesCS3D02G112800.1	R	66,889,410-66,894,969	932	237	8	8	Chromosome 3D: 66,889,410	Peptidyl-prolyl cis-trans isomerase
22	*TaCYP*22	TraesCS3D02G159000	1	TraesCS3D02G159000.1	F	128,439,351-128,445,708	1,160	240	6	6	Chromosome 3D: 128,439,351	Peptidyl-prolyl cis-trans isomerase
23	*TaCYP*23	TraesCS3D02G211900	1	TraesCS3D02G211900.1	R	283,318,822-283,328,008	1894	499	11	10	Chromosome 3D: 2,83,318,822	No description
24	*TaCYP*24	TraesCS4A02G045200	1	TraesCS4A02G045200.1	R	37,302,555-37,306,196	2,144	590	11	11	Chromosome 4A: 37,302,555	No description
25	*TaCYP*25	TraesCS4A02G064400	1	TraesCS4A02G064400.1	R	60,974,303-60,975,739	1,437	478	1	1	Chromosome 4A: 60,974,303	No description
26	*TaCYP*26	TraesCS4A02G168400	2	TraesCS4A02G168400.1	R	420,504,006-420,530,458	1,112	237	8	8	Chromosome 4A: 420,504,006	Peptidyl-prolyl cis-trans isomerase
27	*TaCYP*27	TraesCS4A02G312600	1	TraesCS4A02G312600.1	F	603,637,101-603,639,737	997	180	4	2	Chromosome 4A: 603,637,101	Peptidyl-prolyl cis-trans isomerase
28	*TaCYP*28	TraesCS4A02G423000	1	TraesCS4A02G423000.1	F	693,279,705-693,280,651	820	160	2	2	Chromosome 4A: 693,279,705	Peptidyl-prolyl cis-trans isomerase
29	*TaCYP*29	TraesCS4B02G001300	2	TraesCS4B02G001300.1	F	807,708-809,435	1,055	303	3	3	Chromosome 4B: 807,708	No description
30	*TaCYP*30	TraesCS4B02G241800	1	TraesCS4B02G241800.1	R	500,286,014-500,287,446	1,401	466	2	2	Chromosome 4B: 500,286,014	No description
31	*TaCYP*31	TraesCS4B02G260100	1	TraesCS4B02G260100.1	F	527,004,752-527,008,628	2,216	590	11	11	Chromosome 4B: 527,004,752	No description
32	*TaCYP*32	TraesCS4B02G378800	1	TraesCS4B02G378800.1	R	660,469,673-660,472,270	1,272	326	5	5	Chromosome 4B: 660,469,673	No description
33	*TaCYP*33	TraesCS4D02G001600	1	TraesCS4D02G001600.1	R	1,202,412-1,204,113	661	179	2	1	Chromosome 4D: 1,202,412	Peptidyl-prolyl cis-trans isomerase
34	*TaCYP*34	TraesCS4D02G153700	1	TraesCS4D02G153700.1	R	196,866,707-196,892,845	824	231	8	8	Chromosome 4D: 196,866,707	Peptidyl-prolyl cis-trans isomerase
35	*TaCYP*35	TraesCS4D02G241400	1	TraesCS4D02G241400.1	R	403,416,216-403,418,273	1964	481	2	1	Chromosome 4D: 403,416,216	No description
36	*TaCYP*36	TraesCS4D02G259800	1	TraesCS4D02G259800.1	F	428,966,229-428,969,978	2,217	591	11	11	Chromosome 4D: 428,966,229	No description
37	*TaCYP*37	TraesCS5A02G328900	1	TraesCS5A02G328900.1	F	537,952,053-537,958,238	1,159	216	7	7	Chromosome 5A: 537,952,053	Peptidyl-prolyl cis-trans isomerase
38	*TaCYP*38	TraesCS5A02G467000	1	TraesCS5A02G467000.1	R	645,128,502-645,131,718	1,023	198	7	7	Chromosome 5A: 645,128,502	Peptidyl-prolyl cis-trans isomerase
39	*TaCYP*39	TraesCS5A02G544800	2	TraesCS5A02G544800.1	F	700,344,860-700,347,373	1,204	323	5	5	Chromosome 5A: 700,344,860	No description
40	*TaCYP*40	TraesCS5B02G329000	1	TraesCS5B02G329000.1	F	512,986,799-512,990,909	1,013	216	7	7	Chromosome 5B: 512,986,799	Peptidyl-prolyl cis-trans isomerase
41	*TaCYP*41	TraesCS5B02G478800	1	TraesCS5B02G478800.1	R	650,340,088-650,343,529	997	198	7	7	Chromosome 5B: 650,340,088	Peptidyl-prolyl cis-trans isomerase
42	*TaCYP*42	TraesCS5D02G334800	2	TraesCS5D02G334800.2	F	424,211,642-424,216,055	1,084	216	7	7	Chromosome 5D: 424,211,642	Peptidyl-prolyl cis-trans isomerase
43	*TaCYP*43	TraesCS5D02G479900	1	TraesCS5D02G479900.1	R	517,754,901-517,758,179	981	198	7	7	Chromosome 5D: 517,754,901	Peptidyl-prolyl cis-trans isomerase
44	*TaCYP*44	TraesCS6A02G068900	1	TraesCS6A02G068900.1	R	37,407,147-37,408,119	973	171	1	1	Chromosome 6A: 37,407,147	Peptidyl-prolyl cis-trans isomerase
45	*TaCYP*45	TraesCS6A02G176900	8	TraesCS6A02G176900.8	F	196,185,812-196,193,890	3069	808	15	13	Chromosome 6A: 196,185,812	No description
46	*TaCYP*46	TraesCS6A02G313700	1	TraesCS6A02G313700.1	R	550,283,429-550,288,586	1,690	406	10	9	Chromosome 6A: 550,283,429	No description
47	*TaCYP*47	TraesCS6A02G405800	1	TraesCS6A02G405800.1	F	611,533,101-611,536,885	3785	247	1	1	Chromosome 6A: 611,533,101	Peptidyl-prolyl cis-trans isomerase
48	*TaCYP*48	TraesCS6A02G405900	1	TraesCS6A02G405900.1	F	611,541,075-611,541,578	504	167	1	1	Chromosome 6A: 611,541,075	Peptidyl-prolyl cis-trans isomerase
49	*TaCYP*49	TraesCS6B02G093100	2	TraesCS6B02G093100.2	R	68,922,518-68,923,420	903	171	1	1	Chromosome 6B: 68,922,518	Peptidyl-prolyl cis-trans isomerase
50	*TaCYP*50	TraesCS6B02G208900	9	TraesCS6B02G208900.2	R	274,206,172-274,214,876	3052	849	15	14	Chromosome 6B: 274,206,172	No description
51	*TaCYP*51	TraesCS6B02G343800	1	TraesCS6B02G343800.1	R	605,553,127-605,557,822	1,680	408	10	9	Chromosome 6B: 605,553,127	No description
52	*TaCYP*52	TraesCS6B02G450300	1	TraesCS6B02G450300.1	F	709,120,233-709,120,892	660	219	1	1	Chromosome 6B: 709,120,233	Peptidyl-prolyl cis-trans isomerase
53	*TaCYP*53	TraesCS6B02G450400	1	TraesCS6B02G450400.1	F	709,133,198-709,133,857	660	219	1	1	Chromosome 6B: 709,133,198	Peptidyl-prolyl cis-trans isomerase
54	*TaCYP*54	TraesCS6D02G066700	1	TraesCS6D02G066700.1	R	32,693,962-32,694,930	969	171	1	1	Chromosome 6D: 32,693,962	Peptidyl-prolyl cis-trans isomerase
55	*TaCYP*55	TraesCS6D02G167200	6	TraesCS6D02G167200.3	R	149,726,520-149,734,503	3035	849	15	14	Chromosome 6D: 149,726,520	No description
56	*TaCYP*56	TraesCS6D02G293100	1	TraesCS6D02G293100.1	R	403,802,512-403,807,347	1838	408	10	9	Chromosome 6D: 403,802,512	No description
57	*TaCYP*57	TraesCS7A02G066500	1	TraesCS7A02G066500.1	F	33,368,317-33,369,426	1,002	160	2	2	Chromosome 7A: 33,368,317	Peptidyl-prolyl cis-trans isomerase
58	*TaCYP*58	TraesCS7A02G175300	1	TraesCS7A02G175300.1	F	128,895,090-128,901,210	1747	379	9	8	Chromosome 7A: 128,895,090	No description
59	*TaCYP*59	TraesCS7A02G277700	1	TraesCS7A02G277700.1	R	291,805,787-291,812,889	2,738	648	15	13	Chromosome 7A: 291,805,787	No description
60	*TaCYP*60	TraesCS7A02G279300	1	TraesCS7A02G279300.1	F	297,941,847-297,944,153	798	164	6	6	Chromosome 7A: 297,941,847	Peptidyl-prolyl cis-trans isomerase
61	*TaCYP*61	TraesCS7A02G286700	4	TraesCS7A02G286700.4	R	336,754,940-336,759,756	1706	423	7	7	Chromosome 7A: 336,754,940	No description
62	*TaCYP*62	TraesCS7A02G410100	2	TraesCS7A02G410100.1	R	596,722,888-596,725,543	984	213	7	7	Chromosome 7A: 596,722,888	Peptidyl-prolyl cis-trans isomerase
63	*TaCYP*63	TraesCS7A02G419600	1	TraesCS7A02G419600.1	R	611,338,150-611,343,930	1,503	406	3	3	Chromosome 7A: 611,338,150	No description
64	*TaCYP*64	TraesCS7A02G469800	1	TraesCS7A02G469800.1	R	666,183,734-666,190,033	2,255	559	14	14	Chromosome 7A: 666,183,734	No description
65	*TaCYP*65	TraesCS7B02G080700	1	TraesCS7B02G080700.1	F	91,122,194-91,128,295	1748	380	9	8	Chromosome 7B: 91,122,194	No description
66	*TaCYP*66	TraesCS7B02G175400	1	TraesCS7B02G175400.1	R	246,511,880-246,519,416	1947	648	13	13	Chromosome 7B: 246,511,880	No description
67	*TaCYP*67	TraesCS7B02G180900	1	TraesCS7B02G180900.1	R	271,297,949-271,317,337	907	164	6	6	Chromosome 7B: 271,297,949	Peptidyl-prolyl cis-trans isomerase
68	*TaCYP*68	TraesCS7B02G199200	3	TraesCS7B02G199200.1	R	357,992,011-357,996,264	1,574	423	7	7	Chromosome 7B: 357,992,011	No description
69	*TaCYP*69	TraesCS7B02G309500	2	TraesCS7B02G309500.1	R	553,630,020-553,632,789	1,016	213	7	7	Chromosome 7B: 553,630,020	Peptidyl-prolyl cis-trans isomerase
70	*TaCYP*70	TraesCS7B02G320200	1	TraesCS7B02G320200.1	R	570,460,914-570,467,305	1,521	409	3	3	Chromosome 7B: 570,460,914	No description
71	*TaCYP*71	TraesCS7B02G371900	1	TraesCS7B02G371900.1	R	637,768,745-637,774,947	2,189	559	14	14	Chromosome 7B: 637,768,745	No description
72	*TaCYP*72	TraesCS7D02G060700	1	TraesCS7D02G060700.1	F	33,051,980-33,058,859	803	160	3	2	Chromosome 7D: 33,051,980	Peptidyl-prolyl cis-trans isomerase
73	*TaCYP*73	TraesCS7D02G176900	1	TraesCS7D02G176900.1	F	129,780,067-129,786,404	1777	375	9	8	Chromosome 7D: 129,780,067	No description
74	*TaCYP*74	TraesCS7D02G277600	1	TraesCS7D02G277600.1	R	266,390,457-266,397,119	2,435	648	15	13	Chromosome 7D: 266,390,457	No description
75	*TaCYP*75	TraesCS7D02G279100	2	TraesCS7D02G279100.2	R	269,709,386-269,718,591	927	154	7	6	Chromosome 7D: 269,709,386	Peptidyl-prolyl cis-trans isomerase
76	*TaCYP*76	TraesCS7D02G283600	3	TraesCS7D02G283600.2	F	295,038,517-295,043,103	1857	431	7	7	Chromosome 7D: 295,038,517	No description
77	*TaCYP*77	TraesCS7D02G403300	2	TraesCS7D02G403300.1	R	520,405,786-520,408,608	1,109	213	7	7	Chromosome 7D: 520,405,786	Peptidyl-prolyl cis-trans isomerase
78	*TaCYP*78	TraesCS7D02G412500	2	TraesCS7D02G412500.1	R	530,916,463-530,922,879	1,450	413	3	3	Chromosome 7D: 530,916,463	No description
79	*TaCYP*79	TraesCS7D02G457200	1	TraesCS7D02G457200.1	R	575,679,950-575,686,042	2,204	559	14	14	Chromosome 7D: 575,679,950	No description
80	*TaCYP*80	TraesCSU02G067400	1	TraesCSU02G067400.1	R	53,423,571-53,435,126	1,189	231	8	8	Chromosome Un: 53,423,571	Peptidyl-prolyl cis-trans isomerase
81	*TaCYP*81	TraesCSU02G129100	1	TraesCSU02G129100	R	110,345,273-110,347,940	1,269	325	5	5	Chromosome Un: 110,345,273	No description

### 3.2 Physical mapping of *TaCYP* genes

**FIGURE 1:**
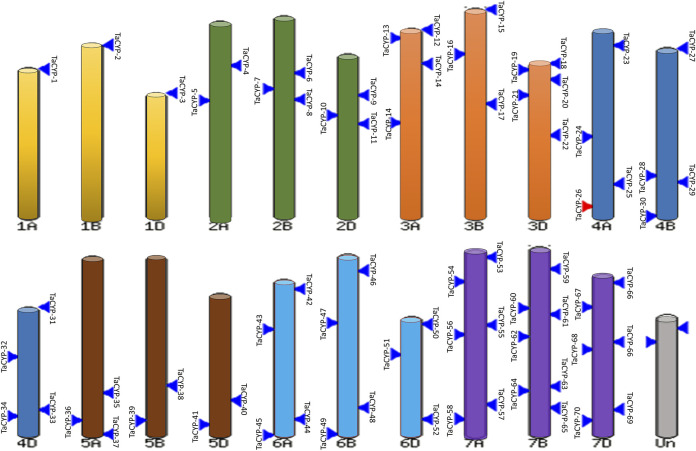
Physical mapping of 81 identified wheat *TaCYP*s based on the image obtained from EnsemblePlants release 47 after BlastN analysis.

Information on the physical mapping of all 81 identified *TaCYP* genes to all 21 wheat chromosomes is depicted in Figure 1. The minimum number of *TaCYP* genes was mapped on homoeologous group 1, and the maximum was located on homoeologous group 7. The range of identity between the three homeologues of each *TaCYP* gene was 70.95%–99.57% for coding sequence, 70.95%–99.57% for amino acid sequence, and 70.95%–99.57% for gene sequence. On the other hand, two *TaCYP* genes (*TaCYP8* and *TaCYP11*) mapped on chromosomes 2B and 2D did not have any homoeologous loci on chromosome 2A (Figure 1 and Table 1).

### 3.3 Gene structure of *TaCYP*s with the distribution of conserved motifs

**FIGURE 2:**
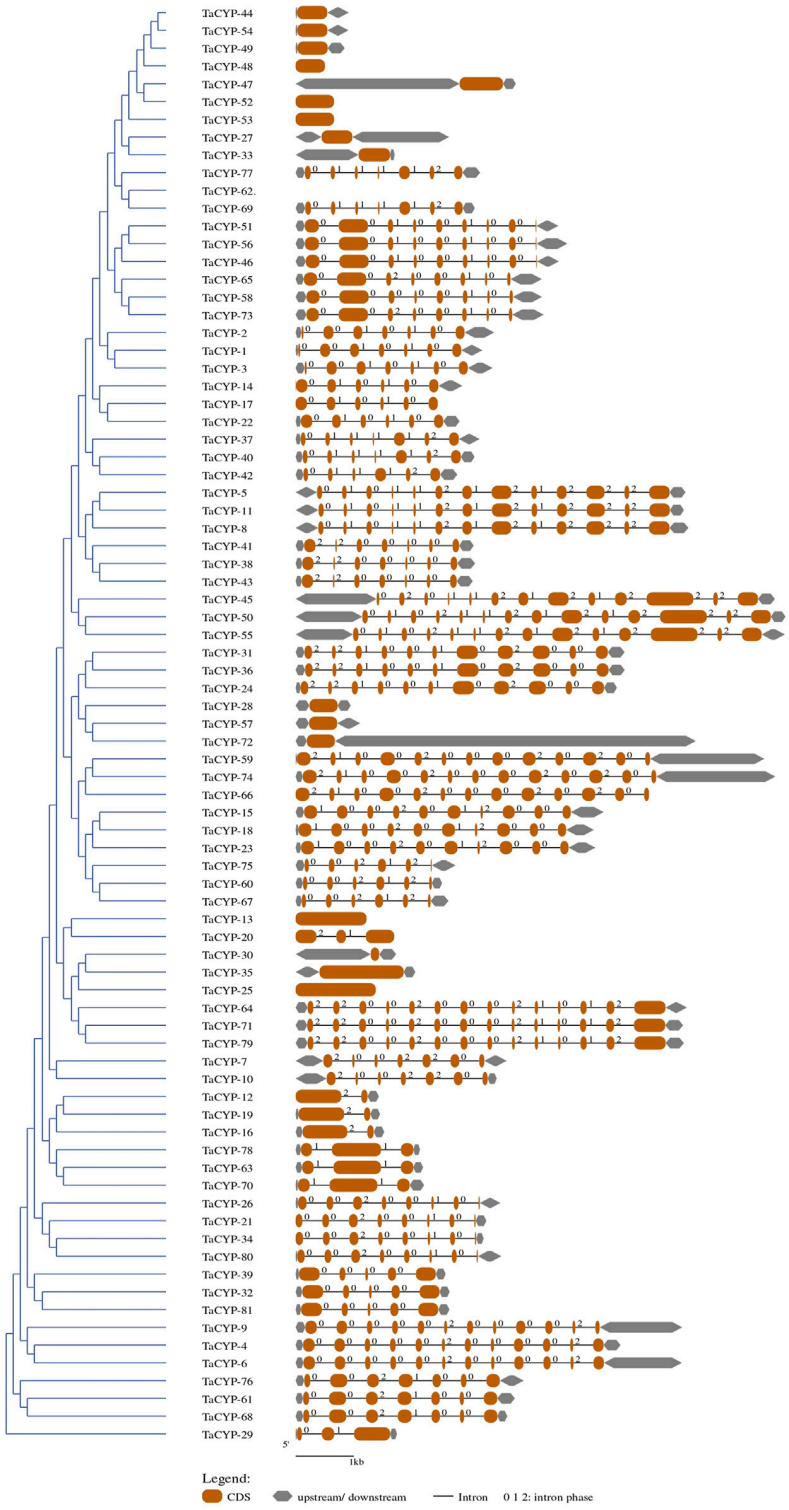
A representative figure depicting the grouping of the predicted gene structure of all 81 *TaCYP* genes identified.

Gene structure predicted using CDS and gDNA sequences of wheat *TaCYP* genes showed diversification between all 81 *TaCYP* genes. The number of exons/introns was highly variable, exon number varied from 1 (*TaCYP*13-3A, *TaCYP*25-4A, *TaCYP*33-4D, *TaCYP*35-4D, *TaCYP*44-6A, *TaCYP*47-6A, *TaCYP*48-6A, *TaCYP*49-6B, *TaCYP*52-6B, *TaCYP*53-6B, and *TaCYP*54-6D) to 14 (*TaCYP*50-6B, *TaCYP*55-6D, *TaCYP*64-7A, *TaCYP*71-7B and *TaCYP*79-7D) (Figure 2). As shown in Figure 2, most *TaCYP* members of a cluster exhibited the same exon/intron boundary patterns, including intron phase, intron number, and exon length.

The MEME analyses of the protein sequences of a set of 81 *TaCYP* genes led to the discovery of 15 distinct conserved motifs (1–15) with a width of 15–41 (Supplementary Figure S1). The location of predicted motifs showed that CYP domains carried a minimum 2 (*TaCYP*32, 39, and 81) to a maximum of 12 (*TaCYP*35, 30, 25, 64, 71, and 79) conserved predicted motifs. Motif 4 was conserved in 77 of 81 identified *TaCYP*s. Out of 81 *TaCYP*s, 27 *TaCYP*s showed a conserved distribution pattern for 8 predicted motifs: motif 11 followed by motifs 3, 1, 5, 6, 2, 4, and 10. Group III members contained maximum number (6–12) of motifs, followed by Group II (7–11 motifs), and group I members (2–7 motifs). Seven motifs (motifs 3, 1, 5, 6, 2, 4, and 10) existed in all members of group II (except *TaCYP45*, which lacked motifs 3 and 1) (Supplementary Figure S1) and Group II (except *TaCYP21*, 26, 34, and 80, those lacked motif 5). The log-likelihood ratio, information content, and relative entropy ranges of the 15 identified motifs ranged from 646-3550, 32-152.8, and 30.2-155.2, respectively (Table 2). The distribution patterns of the 15 identified conserved motifs among the *TaCYPs* are presented in Supplementary Figure S1.

**TABLE 2:** Details of the discovered motif (MEME).

S. No.	Discovered motif	Log likelihood ratio	Information content	Relative entropy	Bayes threshold
1	YYKGSSFHRVIKGFMIQGGDF	2,946	65.8	66.4	8.8
2	NAGPNTNGSQFFITTVPTPWL	2,796	58.8	58.5	10.7
3	TPAGRIVIELYGDVVPKTAENFRALCTGE	3550	68.7	64.8	8.4
4	DGKHVVFGRVVEGMD	2043	39.7	36.8	8.7
5	GTGGESIYGGKFEDE	1775	47.2	43.4	8.8
6	NFKLKHTGPGTLSMA	1769	39.9	38.1	10.1
7	DRPKKDVVILDCGEL	1,442	32	30.2	8.5
8	TGDSLCYAFIAFEEKEGCEKAFFKMGNALIDLRRIDVDFEQ	1,340	120	113.7	11.4
9	AAAAAAAPAAAAAQSPVTPKVFFDVSIGG	1,208	65.8	60.1	10.5
10	WWIEAVDSAKAFGNENFKKHDYKKALRKYRKALRYLDVCWE	878	143.6	140.8	12.3
11	DNVLFVCKLNPVTQDEDLYTIFSRFGTVT	589	109	106.3	11.7
12	CGAPDHIARDCDQGGEKKNKAPBYVLKDENTQRGGNNRRSY	820	152.8	147.8	12.4
13	QLAELIPENSPJGKPRDEIAEERLEDTWV	773	85.1	79.7	10.8
14	FQHALDLEPNDGGIKRELAAAKKKISBRRBKERKAYAKMFZ	646	165.2	155.2	10.9
15	PLDETVDPGQLEELIRSKEAHANAVIQISVGLIPBAEVKPP	977	109.9	100.7	9.8

### 3.4 Phylogenetic analysis

**FIGURE 3:**
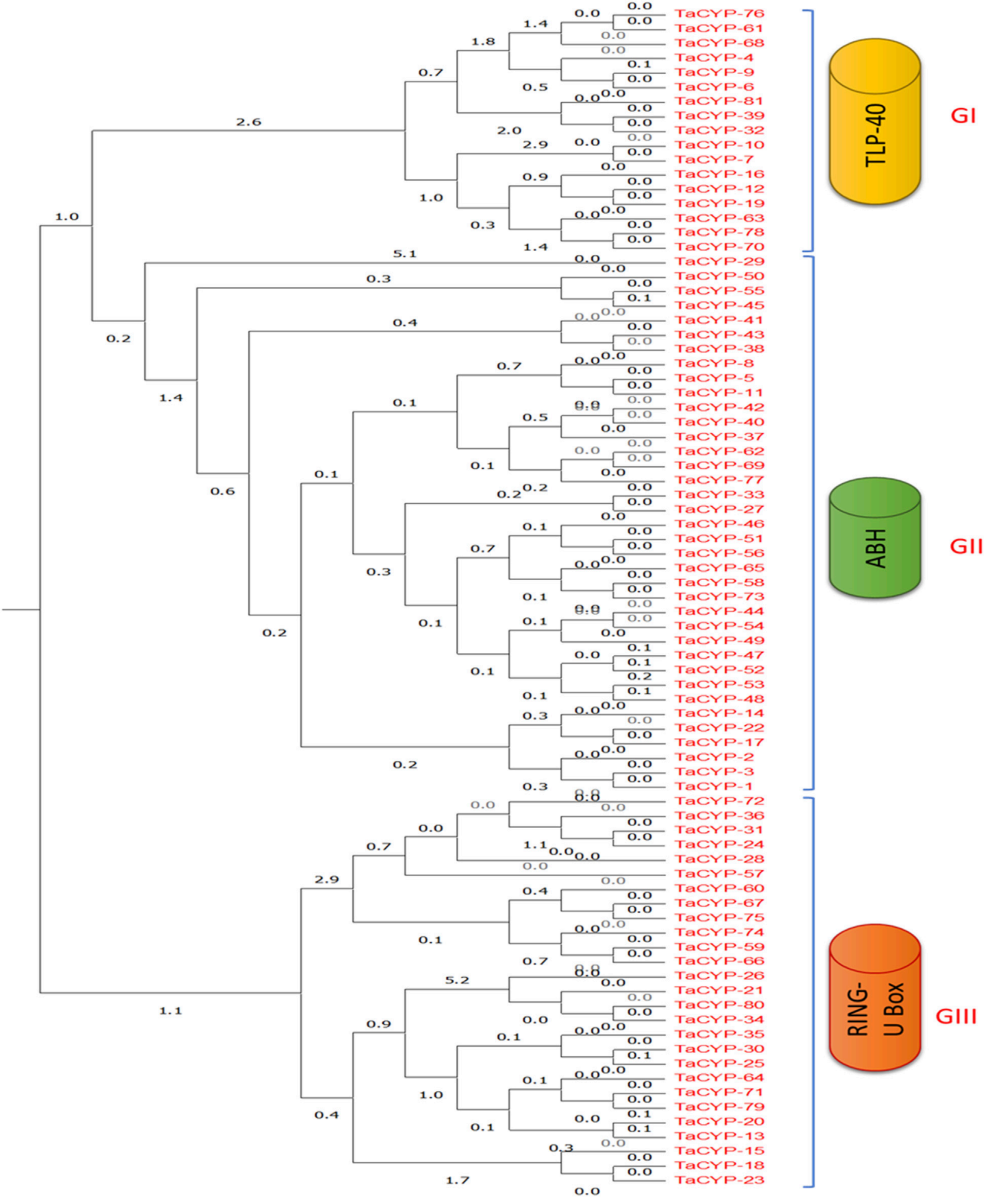
Phylogenetic classification and grouping of all 81 TaCYP proteins with conserved protein domains into three groups: GI, GII, and GIII. GI contains all the members with the TLP-40 domain, GII clusters all members containing the ABH domain, and GIII contains all TaCYPs with the Ring U Box domain.

Phylogenetic analysis using an unrooted maximum likelihood algorithm revealed the clustering of 81 TaCYP proteins into three different groups based on their conserved domains. All TaCYP proteins carry a highly conserved CLD (cyclophilin-like domain) domain with three variants; namely, TLP-40, ABH, and Ring U-Box (Figure 3). For instance, 17 TaCYP proteins (out of 81) that contained the TLP-40 domain were clustered into group I; 37 TaCYP proteins with the ABH domain were clustered into group II, and the remaining 27 TaCYP proteins, which included the ring U box domain, were clustered into group III. Group II was the largest group, with the maximum number of TaCYP members (45.67%). The phylogenetic relationship among the identified TaCYP proteins is given in Figure 3. The phylogenetic relationship between the identified TaCYP proteins and the earlier CYPs reported in other plants is shown in Supplementary Figure S2. Domain analysis of wheat TaCYP proteins and CYP proteins from other crops revealed that all the clustered CYP proteins in the phylogenetic tree carried a conserved domain CSA_PPIASE_2.

### 3.5 Network of miRNAs targeting *TaCYP*s

Network analysis revealed the multiplicity behavior of miRNAs, *i.e.*, one miRNA can target more than one member of the *TaCYP* gene family (Supplementary Figure S3). For instance, tae-miR1127a targets four *TaCYP* genes (*TaCYP*36, *TaCYP*41, *TaCYP*67, and *TaCYP*70), tae-miR1137a targets two *TaCYP* genes (*TaCYP*24 and *TaCYP*64), and tae-miR1130a targets five *TaCYP* genes (*TaCYP*61, *TaCYP*62, *TaCYP*43, *TaCYP*76, and *TaCYP*81). Similarly, one member of *TaCYP* gene is a target for more than one miRNA, such as *TaCYP24,* targeted by three miRNAs: tae-miR1128, tae-miR1137a, and tae-miR1137b-5p (Supplementary Table S1).

### 3.6 *In silico* expression analysis under biotic stress

**FIGURE 4:**
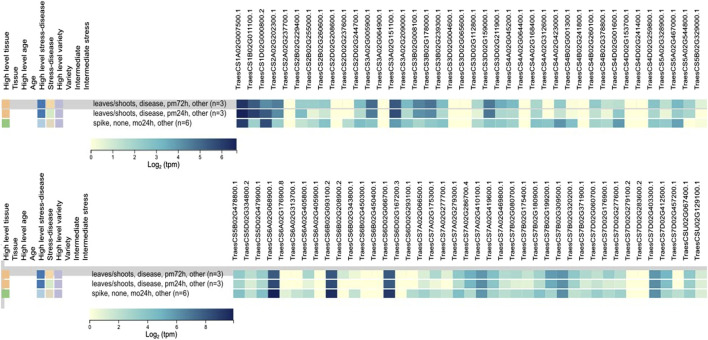
Expression analysis of 81 *TaCYP* genes under abiotic/biotic stress conditions retrieved from the expVIP database using RNA-Seq data.

*In silico* expression analysis of 81 *TaCYP* genes revealed significant expression changes due to infection with powdery mildew. Out of 81 *TaCYP* genes, only three homoeologous transcripts (*TaCYP*44, located on 6A; *TaCYP*49, located on 6B; and *TaCYP*54, located on 6D) showed high expression (8.65–10.37 tpm) against foliar disease infection with powdery mildew at three spans of inoculation (24 and 72 HAI). (Figure 4). The relative expression of each *TaCYP* gene is presented as a heatmap generated from the relative abundance of transcripts (per 10 million reads) for each gene.

### 3.7 Gene expression using qRT–PCR analysis

**FIGURE 5:**
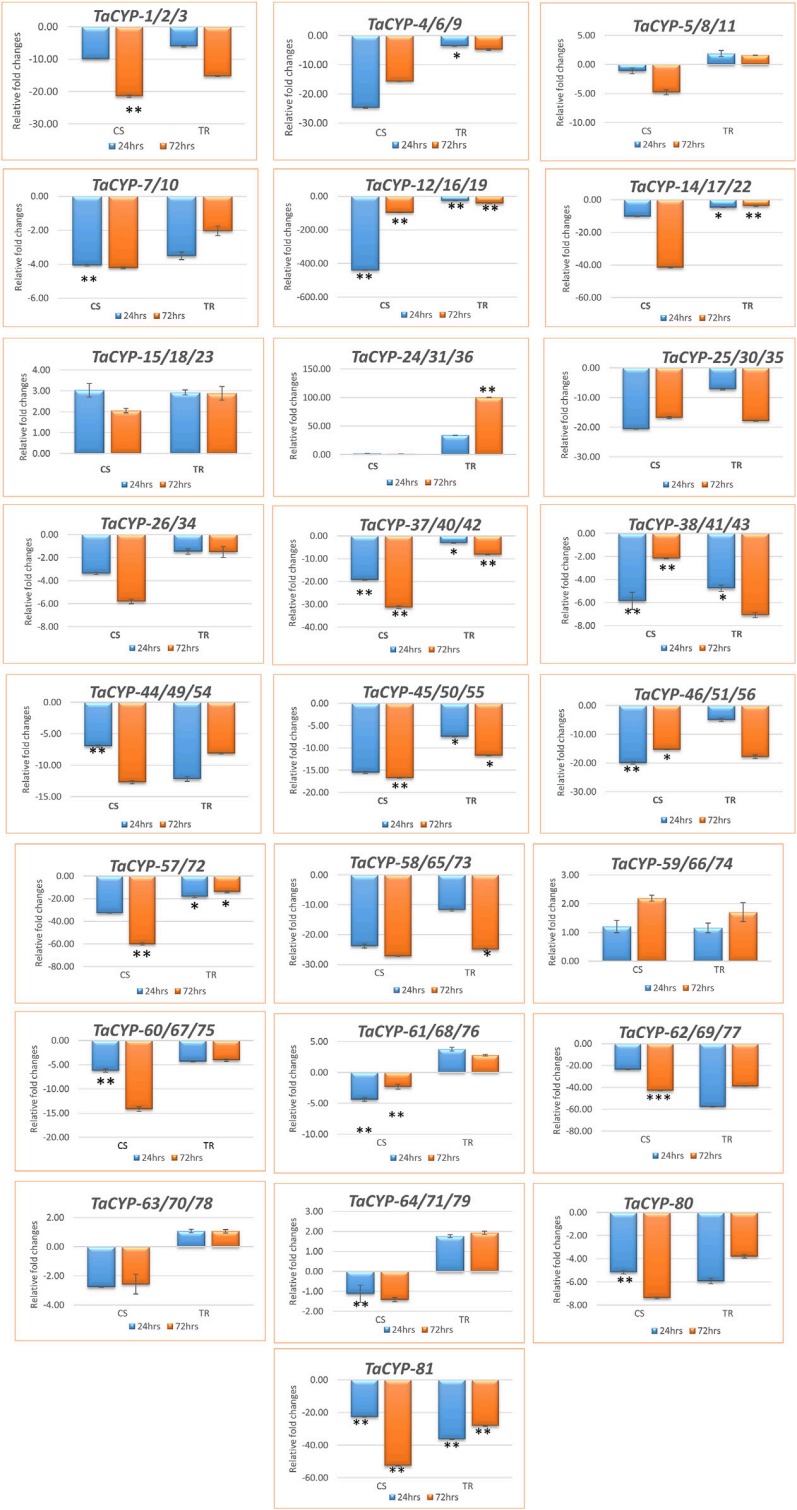
Expression profiling of 81 *TaCYP* genes in leaf rust-resistant genotype transfer (TR) and susceptible genotype Chinese spring (CS) after inoculation with leaf rust pathogens (race 77-5). The mean value of six replicates, with error bars indicating standard error (SE), is presented. SE. Significant changes (based on paired *t* test) in gene expression are indicated by * (*p* > 0.05), **(*p* > 0.01) or & ***(*p* > 0.001).

A total of 25 primers associated with 81 *TaCYP* genes were selected for qRT–PCR analysis based on the grouping of the 81 *TaCYP* genes into 8 groups (seven groups for chromosome 1 to chromosome 7 and one group for sequences with unknown genomic locations). Twenty-three (23) out of the 25 genes belonged to the 7 homeologous groups, whereas 2 genes belonged to unknown chromosomes (for details, see Supplementary Table S2). Eighteen (18) out of the above 25 TaCYPs primers [TaCYP-1 (associated with *TaCYP*1/2/3), 2 (associated with *TaCYP*4/6/9, 4 (associated with *TaCYP*7/10), 5 (associated with *TaCYP*12/16/19), 6 (associated with *TaCYP*14/17/22), 9 (associated with *TaCYP*25/30/35), 10 (associated with *TaCYP*26/34), 11 (associated with *TaCYP*37/40/42), 12 (associated with *TaCYP*38/41/43), 13 (associated with *TaCYP*44/49/54), 14 (associated with *TaCYP*45/50/55), 15 (associated with *TaCYP*46/51/56), 16 (associated with *TaCYP*57/72), 17 (associated with *TaCYP*58/65/73), 19 (associated with *TaCYP* 60/67/75), 21 (associated with *TaCYP*62/69/77), 24 (associated with *TaCYP*80), and 25 (associated with *TaCYP*81) were downregulated in both the contrasting genotypes, whereas three TaCYP primers associated with genes *TaCYP*15/18/23, *TaCYP*24/31/36, and *TaCYP59*/66/74 were upregulated in both genotypes under the disease conditions. However, four TaCYP primers associated with genes *TaCYP*5/8/11, *TaCYP61/68/76*, *TaCYP63/70/78*, and *TaCYP64/71/79* showed significant upregulation in resistant lines. Furthermore, *TaCYP24/31/36* showed maximum upregulation (∼100 FC) in the resistant line compared to the control (Figures 5A,B).

### 3.8 Physiochemical properties

Details of the estimated values of different physiological parameters are given in Table 3. All the selected *TaCYP*s varied for the calculated values, such as the isoelectric points (pIs) from 4.5089 (*TaCYP13*) to 107.231 (*TaCYP77*), the molecular weights (Mws) from 102.434 (*TaCYP19*) to 115.157 g/mol (*TaCYP71*), the theoretical pI from 4.73 (*TaCYP13*) to 12.05 (*TaCYP29*), the instability index (II) from 14 (*TaCYP*57) to 106.61 (*TaCYP*45), the aliphatic index from 40.28 (*TaCYP*45) to 100.04 (*TaCYP*7), and the predicted GRAVY score from −1.44 (*TaCYP*45) to 0.197 (*TaCYP*12). Out of 81 TaCYP proteins, 46 proteins (58%) had a stable nature, while the remaining 35 proteins (42%) were unstable at the sequence level.

**TABLE 3:** Details of 81 *TaCYP* proteins, including average residue weight g/mol, charge, isoelectric point, molecular weight, theoretical PI, instability index, aliphatic index, grand average of hydropathicity (GRAVY) and stability.

Gene	Ave. Residue weight g/mol	Charge	Isoelectric point	Molecular weight g/mol	Theoretical pI	Instability index (II)	Aliphatic index	Grand average of hydropathicity (GRAVY)	Stable
*TaCYP*1	105.884	8.5	9.8314	25,941.5	9.4	35.81	77.63	−0.15	Yes
*TaCYP*2	105.679	9.5	10.1213	25,891.4	9.59	32.44	76.86	−0.151	Yes
*TaCYP*3	105.745	8.5	9.8314	25,907.45	9.4	31.89	77.63	−0.124	Yes
*TaCYP*4	106.099	−1	6.1784	6.1784	5.97	44.11	88.1	−0.11	No
*TaCYP*5	109.834	77	11.083	70,623.28	10.43	66.01	45.26	−1.186	No
*TaCYP*6	106.233	−0.5	6.3737	48,760.75	6.07	44.62	89.59	−0.106	No
*TaCYP*7	112.486	3.5	7.5129	26,209.26	7.07	43.7	100.04	−0.155	No
*TaCYP*8	109.873	75	11.1222	69,769.11	10.46	66.36	41.69	−1.268	No
*TaCYP*9	107.571	4	7.748	45,610.31	7.64	46.05	87.48	−0.136	No
*TaCYP*10	112.336	4	7.5526	26,174.18	7.1	37.92	97.51	−0.192	Yes
*TaCYP*11	110.251	80.5	11.1652	70,119.76	10.51	66.93	44.23	−1.225	No
*TaCYP*12	102.563	12	10.2688	30,256.13	9.74	37.63	97.97	0.197	Yes
*TaCYP*13	111.212	−23.5	4.5089	45,040.97	4.73	39.12	71.46	−0.59	Yes
*TaCYP*14	107.222	3.0	7.485	25,733.2	7.52	34.12	75.13	−0.173	Yes
*TaCYP*15	114.085	−1.5	6.3203	56,472.30	6.01	50.92	68.18	−1.018	No
*TaCYP*16	102.803	12	10.2688	29,915.70	9.74	39.9	96.29	0.158	Yes
*TaCYP*17	106.950	4	7.8016	25,668.11	8.08	39.21	73.08	−0.219	Yes
*TaCYP*18	114.062	−5.5	5.7607	57,030.8	5.69	50.34	68.28	−1.033	No
*TaCYP*19	102.434	12	10.2688	30,218.03	9.74	39.67	96.98	0.178	Yes
*TaCYP*20	112.850	−18	4.5537	37,691.94	4.76	37.56	74.73	−0.585	Yes
*TaCYP*21	112.119	3.5	7.2070	26,572.28	6.76	49.88	79.45	−0.241	No
*TaCYP*22	107.076	4.0	7.7611	25,698.19	8.04	36.35	73.5	−0.196	Yes
*TaCYP*23	114.18	−5.5	5.7586	56,975.62	5.68	48.79	68.22	−1.044	No
*TaCYP*24	109.785	9.5	7.5797	64,773.06	7.29	28.33	67.17	−0.563	Yes
*TaCYP*25	114.223	−7.5	5.5357	5.5357	5.5	39.02	69.14	−0.815	Yes
*TaCYP*26	112.297	5.5	8.2661	26,614.44	8.36	52.05	81.9	−0.265	No
*TaCYP*27	104.669	5	8.4889	18,840.48	8.67	18.9	69.33	−0.172	Yes
*TaCYP*28	107.816	4.5	7.9629	17,250.54	7.85	14.77	72.56	−0.224	Yes
*TaCYP*29	106.897	41.5	12.5473	32,389.69	12.05	89.55	48.88	−0.532	No
*TaCYP*30	113.902	−4.5	6.0702	53,078.52	5.75	41.66	63.82	−0.96	No
*TaCYP*31	109.938	11.5	7.9467	64,863.22	7.96	29.18	67	−0.568	Yes
*TaCYP*32	108.051	11.5	8.6208	35,224.57	8.94	56.07	84.23	−0.043	No
*TaCYP*33	104.901	1	6.8452	18,777.29	6.41	20.17	69.22	−0.141	Yes
*TaCYP*34	111.634	4	7.4773	25,787.39	7.09	46.45	82.77	−0.184	No
*TaCYP*35	114.776	0	6.5051	55,207.15	6.08	42.02	65.28	−0.931	No
*TaCYP*36	109.846	9.5	7.5797	64,919.24	7.29	28.95	67.38	−0.559	Yes
*TaCYP*37	109.055	5	8.4141	23,555.86	8.42	25.62	80.74	−0.237	Yes
*TaCYP*38	108.003	5	8.1615	21,384.52	8.47	25.55	72.42	−0.158	Yes
*TaCYP*39	107.588	9	8.3595	34,750.97	8.72	50.03	84.98	−0.047	No
*TaCYP*40	108.939	4	7.9158	23,530.81	7.77	26.83	82.55	−0.198	Yes
*TaCYP*41	107.790	5	8.1615	21,342.49	8.47	22.63	73.43	−0.127	Yes
*TaCYP*42	108.939	4	7.9158	23,530.81	7.77	26.83	82.55	−0.198	Yes
*TaCYP*43	107.932	5	8.1615	21,370.5	8.47	25.98	71.92	−0.159	Yes
*TaCYP*44	107.550	5.5	8.2502	18,391.07	8.53	18.09	66.02	−0.202	Yes
*TaCYP*45	112.423	120	12.0339	90,837.67	11.51	106.61	40.28	−1.44	No
*TaCYP*46	108.95	−6.5	5.2653	44,233.73	5.42	23.06	69.26	−0.468	Yes
*TaCYP*47	109.049	11	8.9479	26,935.05	9.13	32.74	78.5	−0.1	Yes
*TaCYP*48	106.086	2	7.2659	17,716.4	6.89	18.35	79.34	0.035	Yes
*TaCYP*49	107.48	5.5	8.2495	18,379.05	8.52	19.22	65.44	−0.213	Yes
*TaCYP*50	111.907	121.5	11.9813	95,009.27	11.45	97.11	46.15	−1.261	No
*TaCYP*51	109.16	−6.5	5.2783	44,537.13	5.43	22.11	71.35	−0.438	Yes
*TaCYP*52	107.49	11.5	9.0669	23,540.34	9.16	35.58	82.28	0.047	Yes
*TaCYP*53	109.612	12.5	9.4164	24,004.97	9.3	26.61	79.13	−0.117	Yes
*TaCYP*54	107.55	5.5	8.2502	18,391.07	8.53	18.09	66.02	−0.202	Yes
*TaCYP*55	111.86	121.5	12.0081	94,969.15	11.48	98.59	46.49	−1.257	No
*TaCYP*56	109.191	−7.5	5.1707	44,550.05	5.36	23.87	69.68	−0.468	Yes
*TaCYP*57	107.841	5	7.9801	17,254.53	7.87	14	72.56	−0.261	Yes
*TaCYP*58	109.94	−0.5	6.4227	41,667.07	6.06	29.67	68.58	−0.504	Yes
*TaCYP*59	112.245	5	6.9378	72,734.47	6.49	43.46	76.39	−0.455	No
*TaCYP*60	110.522	5	8.4983	18,125.59	8.55	28.3	71.89	−0.456	Yes
*TaCYP*61	109.323	−13.5	4.5823	46,243.51	4.85	41.13	92.17	−0.217	No
*TaCYP*62	107.297	12	10.1576	22,854.31	9.58	19.38	76.48	−0.146	Yes
*TaCYP*63	107.883	13	8.493	43,800.34	8.77	51.66	72.86	−0.349	No
*TaCYP*64	114.845	−4	6.1908	6.1908	5.85	49.01	55.12	−1.193	No
*TaCYP*65	109.911	0.5	6.5905	41,766.25	6.2	30.5	69.42	−0.486	Yes
*TaCYP*66	112.277	4.5	6.8711	72,755.45	6.42	42.51	76.39	−0.466	No
*TaCYP*67	110.492	4.5	8.2803	18,120.62	8.43	26.26	71.28	−0.45	Yes
*TaCYP*68	109.493	−13.5	4.5854	46,315.62	4.86	41.99	92.39	−0.209	No
*TaCYP*69	107.297	12	10.1576	22,854.31	9.58	19.38	76.48	−0.146	Yes
*TaCYP*70	108.171	14	8.8576	44,241.8	9.01	49.85	75.4	−0.35	No
*TaCYP*71	115.157	−3.5	6.2187	64,372.61	5.87	49.93	55.46	−1.202	No
*TaCYP*72	107.753	5	7.9801	17,240.5	7.87	14.77	72.56	−0.261	Yes
*TaCYP*73	110.248	0	6.5065	41,342.82	6.14	29.65	70.35	−0.479	Yes
*TaCYP*74	112.125	3	6.7563	72,657.28	6.31	43.04	76.23	−0.467	No
*TaCYP*75	109.949	4.5	8.1469	16,932.17	8.36	25.68	62.66	−0.501	Yes
*TaCYP*76	109.489	−13	4.6255	47,189.59	4.89	40.3	92.27	−0.202	No
*TaCYP*77	107.231	12	107.231	22,840.28	9.58	19.78	76.01	−0.157	Yes
*TaCYP*78	108.466	13	8.5808	44,796.47	8.83	48.86	75.11	−0.353	No
*TaCYP*79	115.334	−6.5	5.89	64,471.61	5.69	52.23	54.6	−1.23	No
*TaCYP*80	111.694	4	7.4774	25,801.42	7.09	47.18	82.77	−0.184	No
*TaCYP*81	107.663	11.5	8.6208	34,990.32	8.94	51.72	86.58	−0.014	No

Prediction of subcellular localization analysis indicated that TaCYP proteins are localized throughout the cell, including different cell organelles. Maximum TaCYP proteins were localized in the nucleus (27 TaCYPs), followed by the extracellular space (13 TaCYPs), cytoplasm (9 TaCYPs), chloroplast thylakoid lumen (8 TaCYPs), organelle membrane (7 TaCYPs), mitochondrial membrane (4 TaCYPs), chloroplast thylakoid membrane (3 TaCYPs), endomembrane system (3 TaCYPs), mitochondrion (2 TaCYPs), chloroplast (2 TaCYPs), chloroplast outer membrane (2 TaCYPs), and plasma membrane (1 TaCYP) (Table 4). *TaCYP* genes located in the nucleus (e.g., *TaCYP5*, *TaCYP*8, *TaCYP* 11, *TaCYP64, TaCYP71,* and *TaCYP79*) showed longer exon–intron architecture (coding exons: 14), while the *TaCYP* genes located in the extracellular space (*TaCYP44, TaCYP49, TaCYP54, TaCYP57, TaCYP72*) and chloroplast thylakoid membrane (*TaCYP12, TaCYP16, TaCYP19*) showed the shortest exon–intron (coding exons: 1 or 2) frame.

**TABLE 4:** Subcellular location of all 81 identified *TaCYP* genes.

Protein accession/ID	GO-id	GO TERM	Score	Features
*TaCYP*1	GO:0009543	chloroplast thylakoid lumen	0.86	CTP
*TaCYP*2	GO:0009543	chloroplast thylakoid lumen	0.86	CTP
*TaCYP*3	GO:0009543	chloroplast thylakoid lumen	0.87	CTP
*TaCYP*4	GO:0009535	chloroplast thylakoid membrane	0.68	CTP,TAH
*TaCYP*5	GO:0005634	Nucleus	1	
*TaCYP*6	GO:0009535	chloroplast thylakoid membrane	0.68	CTP,TAH
*TaCYP*7	GO:0009507	Chloroplast	0.78	
*TaCYP*8	GO:0005634	Nucleus	1	
*TaCYP*9	GO:0009535	chloroplast thylakoid membrane	0.63	CTP,TAH
*TaCYP*10	GO:0012505	endomembrane system	0.78	TAH
*TaCYP*11	GO:0005634	Nucleus	1	
*TaCYP*12	GO:0009543	chloroplast thylakoid lumen	0.72	CTP
*TaCYP*13	GO:0005634	Nucleus	1	
*TaCYP*14	GO:0005739	Mitochondrion	0.97	MTP
*TaCYP*15	GO:0005634	Nucleus	1	
*TaCYP*16	GO:0009543	chloroplast thylakoid lumen	0.71	CTP
*TaCYP*17	GO:0009543	chloroplast thylakoid lumen	0.86	CTP
*TaCYP*18	GO:0005634	Nucleus	1	
*TaCYP*19	GO:0009543	chloroplast thylakoid lumen	0.71	CTP
*TaCYP*20	GO:0005634	Nucleus	1	
*TaCYP*21	GO:0012505	endomembrane system	0.69	TAH
*TaCYP*22	GO:0009543	chloroplast thylakoid lumen	0.86	CTP
*TaCYP*23	GO:0005634	Nucleus	1	
*TaCYP*24	GO:0005737	Cytoplasm	0.7	
*TaCYP*25	GO:0005634	Nucleus	1	
*TaCYP*26	GO:0012505	endomembrane system	0.75	TAH
*TaCYP*27	GO:0005634	Nucleus	1	
*TaCYP*28	GO:0005615	extracellular space	1	
*TaCYP*29	GO:0005739	Mitochondrion	0.59	MTP
*TaCYP*30	GO:0005615	extracellular space	0.7	
*TaCYP*31	GO:0005737	Cytoplasm	0.7	
*TaCYP*32	GO:0031090	organelle membrane	0.73	TAH
*TaCYP*33	GO:0005634	Nucleus	1	
*TaCYP*34	GO:0005615	extracellular space	0.99	SP
*TaCYP*35	GO:0005634	Nucleus	1	
*TaCYP*36	GO:0005737	Cytoplasm	0.7	
*TaCYP*37	GO:0005615	extracellular space	0.89	SP
*TaCYP*38	GO:0005634	Nucleus	1	
*TaCYP*39	GO:0031090	organelle membrane	0.57	TAH
*TaCYP*40	GO:0005615	extracellular space	0.78	SP
*TaCYP*41	GO:0005634	Nucleus	1	
*TaCYP*42	GO:0005615	extracellular space	0.78	SP
*TaCYP*43	GO:0005634	Nucleus	1	
*TaCYP*44	GO:0005615	extracellular space	0.58	
*TaCYP*45	GO:0005634	Nucleus	1	
*TaCYP*46	GO:0005737	Cytoplasm	0.7	
*TaCYP*47	GO:0009507	Chloroplast	1	
*TaCYP*48	GO:0005615	extracellular space	0.7	
*TaCYP*49	GO:0005615	extracellular space	0.56	
*TaCYP*50	GO:0005634	Nucleus	1	
*TaCYP*51	GO:0005737	Cytoplasm	0.7	
*TaCYP*52	GO:0005886	plasma membrane	0.76	TAH
*TaCYP*53	GO:0031090	organelle membrane	0.71	TAH
*TaCYP*54	GO:0005615	extracellular space	0.58	
*TaCYP*55	GO:0005634	Nucleus	1	
*TaCYP*56	GO:0005737	Cytoplasm	0.7	
*TaCYP*57	GO:0005615	extracellular space	0.92	
*TaCYP*58	GO:0005737	Cytoplasm	0.7	
*TaCYP*59	GO:0005634	Nucleus	1	
*TaCYP*60	GO:0005634	Nucleus	1	
*TaCYP*61	GO:0031966	mitochondrial membrane	0.71	MTP,TAH
*TaCYP*62	GO:0031090	organelle membrane	0.88	TAH
*TaCYP*63	GO:0031966	mitochondrial membrane	0.56	MTP,TAH
*TaCYP*64	GO:0005634	Nucleus	1	
*TaCYP*65	GO:0005737	Cytoplasm	0.7	
*TaCYP*66	GO:0005634	Nucleus	1	
*TaCYP*67	GO:0005634	Nucleus	1	
*TaCYP*68	GO:0031966	mitochondrial membrane	0.73	MTP,TAH
*TaCYP*69	GO:0031090	organelle membrane	0.88	TAH
*TaCYP*70	GO:0009707	chloroplast outer membrane	0.7	CTP,TAH
*TaCYP*71	GO:0005634	Nucleus	1	
*TaCYP*72	GO:0005615	extracellular space	0.87	
*TaCYP*73	GO:0005737	Cytoplasm	0.7	
*TaCYP*74	GO:0005634	Nucleus	1	
*TaCYP*75	GO:0005634	Nucleus	1	
*TaCYP*76	GO:0031966	mitochondrial membrane	0.64	MTP,TAH
*TaCYP*77	GO:0031090	organelle membrane	0.87	TAH
*TaCYP*78	GO:0009707	chloroplast outer membrane	0.6	CTP,TAH
*TaCYP*79	GO:0005634	Nucleus	1	
*TaCYP*80	GO:0005615	extracellular space	0.99	SP
*TaCYP*81	GO:0031090	organelle membrane	0.67	TAH

### 3.9 Homology modeling

**FIGURE 6:**
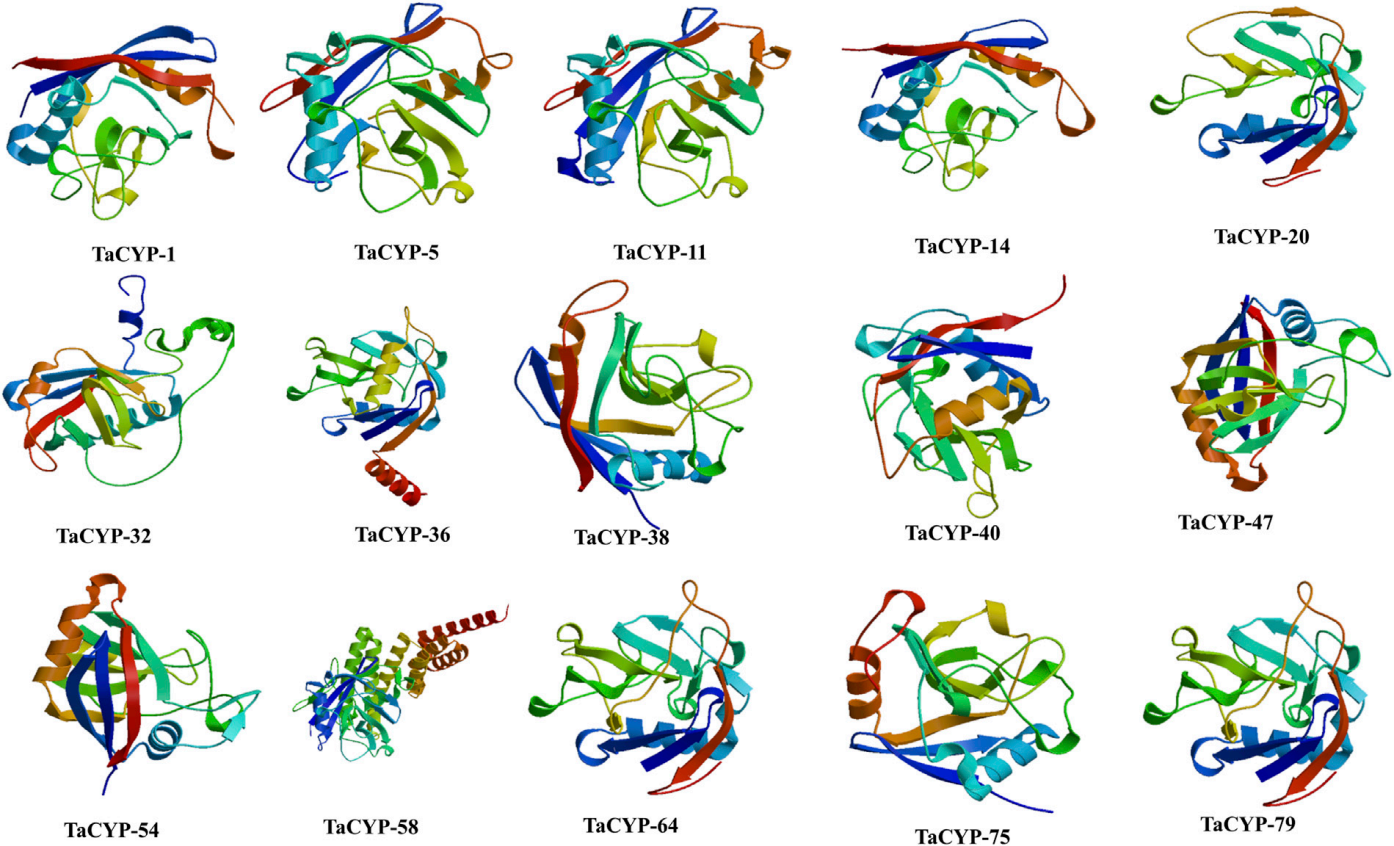
3D structures of 15 representative proteins simulated using the SWISS-MODEL server.

The 3D structures of fifteen (15) representative TaCYP proteins were modeled based on the homology modeling approach. Modeled 3D structures of TaCYP proteins shared a high similarity up to 100% with template structures. The obtained percentage of protein similarity was adequate for annotating protein 3D structures that were predicted using an automated Swiss-Model server. As per the homology modeling method rule, a good protein model should be more than 30% similar to the template structure (Kumar et al., 2019). Modeled 3D structures were further interactively visualized in CPK by UCSF CHIMERA (Figure 6). Calculated 3D structures of fifteen (15) representative proteins depict <1 Å RMSD values for suitable template structures upon superposition.

Ramachandran plot calculation is crucial to evaluate the quality of protein 3D structure and trend. As evident from Supplementary Figure S4 and Supplementary Table S3, the evaluated Ramachandran plots of torsion angles for phi (Φ) and psi (Ψ) revealed the excellent geometry of the predicted 3D structures of TaCYPs. The calculated Ramachandran plots of TaCYPs showed up to 90.2% residues in most favored regions and up to 21.6% in additional allowed regions. In contrast, up to 2.6 residues in generously allowed regions follow the suitable quality parameters of the PROCHECK algorithm (Supplementary Figure S4). The fruitful utilization of the Ramachandran plot has been demonstrated in several recent findings (Arnold et al., 2006; Kumar et al., 2016; 2018b).

### 3.10 Accumulation of ROS

**FIGURE 7:**
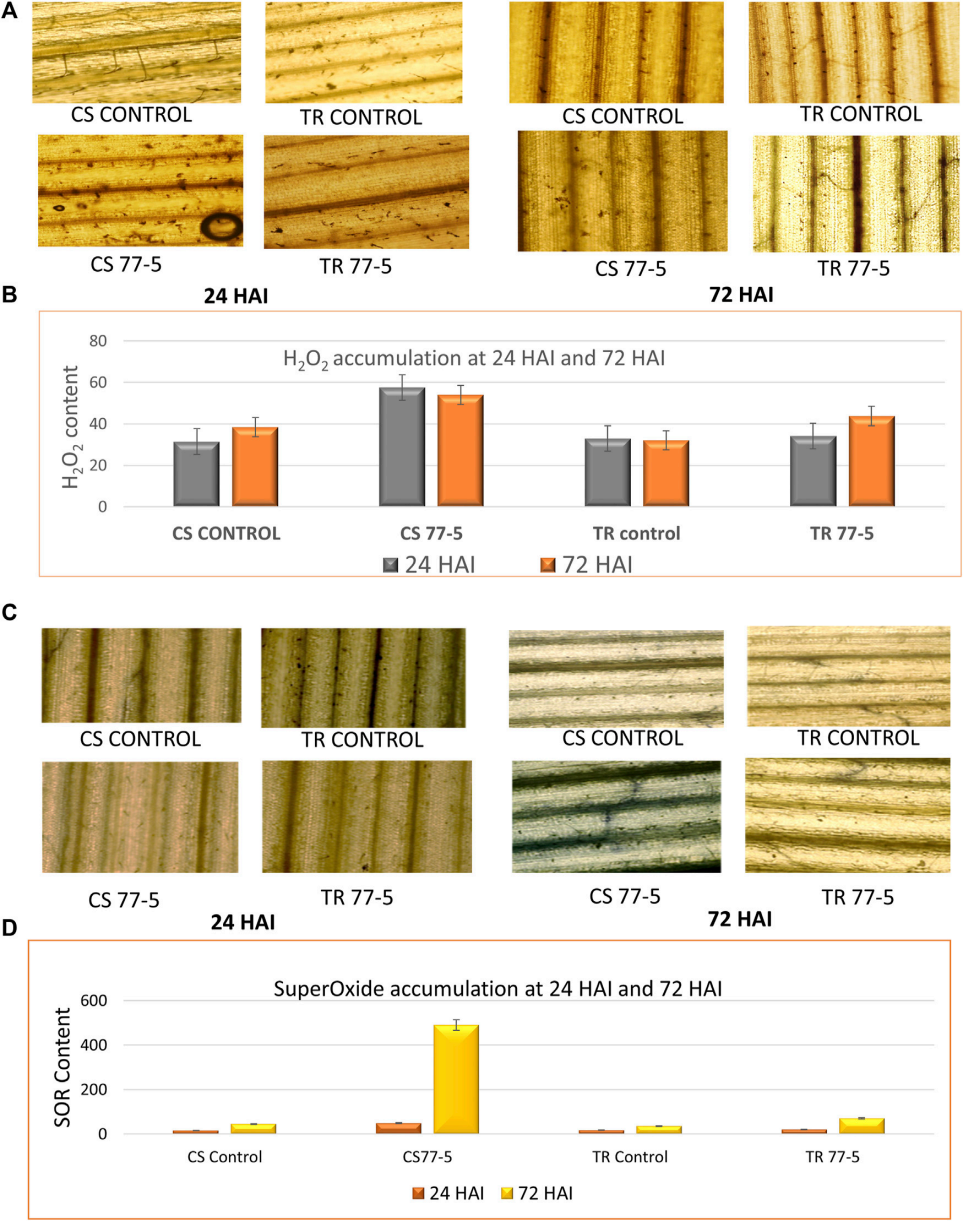
Effects of leaf rust pathogen on the localization and content of reactive oxygen species, hydrogen peroxide **(A,B)** and superoxide radical **(C,D)**. CS; Chinese spring and TR; Transfer.

The results suggest an ROS burst, as indicated by the localization and accumulation of ROS [SOR and H_2_O_2_] contents in wheat seedlings. The presence of H_2_O_2_ was confirmed via the appearance of the brown-colored product, while the development of dark blue colour indicated the presence of SOR (Figure 7). The spectrophotometric assay and tissue localisation indicates more SOR and H_2_O_2_ in CS w.r.t. TR at 24 and 72 HAI (Figure 7). The correlation heatmap showed that the accumulation of H_2_O_2_ and SOR positively correlated with the *TaCYP* genes during the span of infections (24HAI and 72 HAI) in CS. On the other hand, the accumulation of H_2_O_2_ showed a negative correlation with the *TaCYP* genes, which showed downregulation during 24 HAI and upregulation with the 72 HAI span of infection (Supplementary Figure S5) in TR.

## 4 Discussion

A significant area of varietal development is the breeding of wheat varieties that are resistant to the rust disease, and continuing research is being carried out in this direction. Understanding the molecular basis of leaf rust resistance will be aided by characterising genes involved in downstream signaling during wheat-leaf rust infection. The allelic information of regulatory genes can then be utilized to create functional markers for leaf rust resistance molecular breeding. In order to develop wheat cultivars resistant to leaf rust, it was planned for the current study to characterise CYP genes on a genome-wide scale and identify important CYP candidate genes that may serve as possible targets for allele mining and functional marker development.

Our initial genome-wide search revealed 81 members of the CYP gene family, and it was discovered that these 81 members were dispersed throughout all 21 bread wheat chromosomes, indicating more sequence divergence. The same number of introns/exons and nearly identical length of CDS/aa displayed by the TaCYPs on the chromosomes of the same homoeologous group can be attributed to the CYP members conservation between homoeologous chromosomes or to major structural rearrangements. This implies that among these known TaCYPs, mutation and selection are evolutionarily conserved (Yu et al., 2022). This is also supported by an earlier study that found intron sites that are likely preserved (ancestral) throughout multiple kingdoms (including animals, plants, and fungi) (Fedorov et al., 2002). The chromosomal locations of all identified *TaCYPs* revealed an intriguing pattern of clustering, with *TaCYPs* clustered on one chromosome and their paralogs clustered on the other. These results indicate segmental duplication, which has been demonstrated to be crucial in the evolutionary analysis of several other gene families (Moore et al., 2015), is also involved in the chromosomal areas harbouring *TaCYP* genes.

Further, the phylogenetic analysis revealed the following interesting findings: 1) In the evolutionary tree, 66 of the 81 TaCYP proteins were clustered into a group of three (22 pairs) and the remaining 15 TaCYPs were placed as a separate branch, 2) multidomain (MD) wheat cyclophilin genes (MD-TaCYPs) were clustered together, and 3) a correlation was observed between the clustering pattern of TaCYPs and their subcellular localization. For example, genes predicted to be found in the chloroplast, nucleus, and mitochondria were found to be divided into three distinct clusters based on their predicted location. Maximum *TaCYP* genes in a specific group in the phylogenetic tree matched exon-intron numbers, intron phases, and other characteristics (Figure 3).

The sub-organelle membrane is the location of the majority of the CYP members clustered with the TLP40 domain. It has been reported that TLP40 (MD) cyclophilins play a significant function in the photosynthetic membrane of chloroplasts by acting as negative regulators of the thylakoid membrane protein phosphatase (Fulgosi et al., 1998; Vener et al., 1999; Olejnik et al., 2021). For instance, they influence the dephosphorylation of a number of crucial proteins in photosystem II, which is engaged in light reactions during photosynthesis (Fulgosi et al., 1998), and hence play a significant role in chloroplast biogenesis and intracellular signalling. Because leaf rust is known to have a major impact on photosynthesis rate and diminish grain production (Yahya et al., 2020), the presence of this crucial domain may help in increasing photosynthesis in wheat varieties that have been affected. Additionally, *AtCYP38* in Arabidopsis is a homolog of TLP40 in spinach (Fulgosi et al., 1998), and in the current study, *AtCYP38* formed a cluster with all CYP genes (with TLP40 domain) located in the sub-organelle membrane.

The CYPs in a particular group also had a similar pattern of conserved motifs. Overall, the CYP contained 15 conserved regulatory motifs, which confirms previous reports for CYPs in Arabidopsis and rice (Romano et al., 2004; Singh et al., 2019). However, some motifs exclusively appeared in a particular group of TaCYP proteins; motifs 9, 13, and 14 were present in the members of group III, and upregulated genes (*TaCYP24*) along with their homeologus genes (*TaCYP31* and *TaCYP36*) contain two copies of motif 14 compared to other genes, which may provide specificity during resistance. The motif distribution among *TaCYP*s suggested that the proteins in the same group perform identical functions (Schaeffer et al., 2016).

The range of the protein instability index, which is variable, may be an indication of the variability in the stability of wheat TaCYP proteins under a variety of environmental conditions, including potential biotic stresses. The degree of thermal stability demonstrated by a protein under a range of stresses is indicated by the aliphatic index derived in the current study for various TaCYP proteins. As a result, proteins with higher values of the aliphatic index may be more thermostable than those with lower values of the aliphatic index (Rashid and Salih, 2022). The aliphatic index of TaCYP proteins in the current study ranged from 40.28 to 100.04, demonstrating that these TaCYP proteins are thermostable under a spectrum of conditions.

Protein stability at the sequence and structure levels play important role in controlling the plant immune system in response to biotic stress (Holt et al., 2005). Most (76 out of 81) of the identified TaCYP proteins had a negative GRAVY value, indicating the hydrophilic nature of the proteins. Only five proteins (TaCYP12, TaCYP16, TaCYP19, TaCYP48, and TaCYP52) show a hydrophobic nature, suggesting a high level of stability of the identified TaCYP proteins. Protein localization results revealed the clustering of most genes in the nucleus (27 TaCYPs), It is not surprising given that the nucleus is where active signaling genes are most frequently targeted (Peng and Gao, 2014; Robles and Quesada 2021). The identified homoeologous *TaCYP* genes shared a similar pattern of exon–intron structure and intron phrase in the same sub cell organelle, e.g., *TaCYP4*(2A)*, TaCYP6* (2B)*,* and *TaCYP9* (2D) are localized in the chloroplast thylakoid membrane; similarly, *TaCYP24*(4A), *TaCYP31*(4B), and *TaCYP36*(4D) are members of subcellular organelle cytoplasm, confirming structural rearrangements or conservation of CYP members between homoeologous chromosomes. The present study revealed that a highly upregulated group of TaCYP genes (*TaCYP24, TaCYP31,* and *TaCYP36*) are localized in the cytoplasm, which also receives support from an earlier study involving the Arabidopsis-*P. syringeae* pathosystem, where the overexpressed *AtCYP19* and *AtCYP57* genes were also localized in the cytoplasm and their overexpression induced resistance against *Pseudomonas syringae* (Pogorelko et al., 2014). Therefore, we believe that the above three upregulated genes (*TaCYP24, TaCYP31,* and *TaCYP36*) in the present study may have a potential role in providing resistance against leaf rust infection; however, future studies involving overexpression or suppression through suitable approaches will lead to a better understanding of the role of these genes during wheat-leaf rust interactions.

When the genes were analysed for expression using qRT-PCR, the amplicon from primer XTaCYP-8 (derived from the genes *TaCYP24*, *TaCYP31*, and *TaCYP36*) located on similar location of homoeologous chromosomes 4A, 4B, and 4D exhibited a significant upregulation (100FC) in the resistant line as compared to the control. Additionally, these were found to be an ortholog of the peptidyl-prolyl cis-trans isomerase gene that has been previously identified in a variety of crops, including rice (*OsCYP65*), Arabidopsis (*AtCYP65*), *Sorghum bicolor* (SORBl3001G466700), *Brassica napus* (BnaC03g48580D), *Hordeum vulgare* (*HORVHr1* (AET4Gv20643700). It is a protein that functions as a RING-type E3 ubiquitin transferase isomerase in the folding, peptidyl-prolyl isomerization, and polyubiquitination of proteins. It has been previously reported that wheat’s E3 ubiquitin ligase participates in the defence response against the Bgt fungus and against salt stress (Li et al., 2014; Zhu et al., 2015).

In addition, a leaf rust-resistant QTL *Lr. ace-4A*, conferring resistance at the seedling stage and tightly linked with the stem rust-resistant QTL *QSr.ace-4A,* has also been identified and mapped on the short arm of chromosome 4A within a QTL interval of 37, 813, 793 bp–581,470,783 bp (Aoun et al., 2019). An *in silico* study revealed that the identified wheat *TaCYP24* is also located at 37,302,555 bp–37,306,196 bp on chromosome 4AS, indicating that the differentially expressed *TaCYP24* gene is a strong candidate or some cis-regulatory element involved during resistance through a leaf rust-resistant QTL (*Lr.ace-4A*) that maps to this region. Earlier, it was also demonstrated that variation in sequences near candidate genes is often responsible for the prominent differences in expression (Mozhui et al., 2008).

The *in silico* experiment filtered out three highly expressed transcripts of homoeologous genes, *TaCYP44, TaCYP49*, and *TaCYP54,* at the leaf disease stage against powdery mildew. The qRT–PCR experiment also showed that the gene associated with primer XTaCYP-13 (designed from the cluster of *TaCYP44, TaCYP49*, and *TaCYP54*) displayed the differential expression in contrasting lines. Further, the TBLASTN confirmed that Arabidopsis ROTAMASE CYCLOPHILIN 1 (*ROC1*) (AtCYP18-3; used as a query sequence in the present study) has three orthologous genes in wheat: *TaCYP44* (6A), *TaCYP49* (6B), and *TaCYP54* (6D). It has been validated that the *AtROC1* modulates the immunity specified by R proteins NLRs, RPM1 and RPS2 and concludes that prolyl-peptidyl isomerase activity is required for immune response regulation (Trupkin et al., 2012; Li et al., 2014). Additionally, it has been confirmed that effector AvrRpt2 is activated by binding of host CYP that results in proper folding of AvrRpt2 by virtue of prolyl isomerization catalyzed by host CYP. Activation of AvrRpt2 leads to the cleavage of RIN4, which further activates RPS2 (R protein) and the subsequent orchestration of defense responses (Day et al., 2005). Therefore, the function of the CYP homeologues on chromosome six can be linked to their involvement in leaf rust resistance.

The miRNA targeting wheat *TaCYP* search resulted in the identification of miR1137 targeting *TaCYP*24. The role of isomiRs of the miR1137 family has also been reported in targeting anthranilate synthase (AS) (Ravichandran et al., 2019), which helps to catalyze the first reaction branching from the AAA pathway (aromatic amino acid pathway of plants, fungi, and bacteria) toward the biosynthesis of tryptophan and has been studied for its role against pathogens and herbivores. An increase in steady-state AS mRNA levels during/after infiltration helps in the production of secondary metabolites and provides resistance against bacterial pathogen infection (Pal and Gardener 2006; Pusztahelyi et al., 2015). In view of the above, it has been suggested that the low expression of miR1137 in resistant varieties results in a higher accumulation of the target gene *TaCYP24*, *TaCYP31*, and *TaCYP36* transcripts*.* Furthermore, miR1137 is downregulated during stripe rust infection in resistant lines (Ramachandran et al., 2020), supporting that the expression of these genes could be regulated through miR1137. However, further study needs to be conducted to explore the detailed role of miR1137 during leaf rust resistance.

ROS production is often the earliest manifestation of the host defense response (Wojtaszek 1997; Dietz et al., 2016; Sewelam et al., 2016). Several studies have suggested that plant-derived ROS generated by membrane-bound Nox and apoplast-secreted peroxidase are involved in the host defense response to cereal rust fungi (Fofana et al., 2007; Dmochowska-Boguta et al., 2013). Our results on SOR localization in response to leaf rust showed maximum accumulation at 72 HAI. In an earlier study, the localization of SOR was observed in the case of the incompatible race but not in the compatible race (Doke, 1983). A recent study demonstrated that Puccinia triticina (Pt) generates ROS, and ROS are critical in the virulence of the wheat leaf rust fungus *Puccinia triticina* (Wang et al., 2020). The upregulated *TaCYP24/31/36* genes also showed maximum expression at 72 HAI. Additionally, an earlier study showed that overexpression of CMPG1–V (in transgenic wheat) provided resistance against powdery mildew in wheat and was associated with an increase in the expression of H_2_O_2_ accumulation (Zhu et al., 2015). Previously, the overexpression of *AtCYP1*9 was reported to be involved in ROS production (Olejnik et al., 2021). The fact that the *TaCYP* genes (*TaCYP24*, *TaCYP31*, and *TaCYP36*) grouped with *AtCYP19* in the current study displayed upregulation at 72 HAI compared to 24 HAI suggests that these genes play a role in the control of ROS during rust infection. On the other hand, the *ROC1*/*AtCYP18-3* orthologous gene in wheat [*TaCYP44* (6A), *TaCYP49* (6B), and *TaCYP54* (6D) showed a downregulated expression pattern], confirming the negative regulation of these *CYP* genes during wheat rust interaction. The correlation of *TaCYP* gene expression and ROS accumulation at 24 HAI and 72 HAI after inoculation in TR and CS indicates a significant association (Supplementary Figures S5A,B). For example, as depicted in the correlation heatmap, the expression of most of the downregulated *TaCYP*s showed a positive correlation with H_2_O_2_ and SOR accumulation in CS and a negative correlation in TR.

## 5 Conclusion

In the present study, we report genome-wide analysis to identify the role of *TaCYP* genes against wheat leaf rust. The *TaCYP*24/31/36 genes located on homoeologous chromosome 4, were maximally upregulated in the leaf rust resistant line compared to the susceptible line and will be potential targets for further validation and molecular breeding approaches. Also the current presents a significant correlation of CYPs gene expression nad and the accumulation of SOR and H_2_O_2_ during leaf rust infection in wheat. The current findings significantly extend previous conclusions about the role of CYP genes and reveal their critical role in minimizing the effect of leaf rust disease in the world’s second most important cereal crop.

## Data Availability

The original contributions presented in the study are included in the article/Supplementary Material, further inquiries can be directed to the corresponding authors.
