# Supplementary material for: Genome-wide characterization and identification of cyclophilin genes associated with leaf rust resistance in bread wheat (Triticum aestivum L.)
Source: Front Genet. 2022 Sep 30;13:972474. doi: 10.3389/fgene.2022.972474 (PMC9561851; doi:10.3389/fgene.2022.972474)
Supplement: Supplementary file 1 [file Table1.DOCX]

**Suppl. Table 1:** List of identified miRNAs targeting wheat TaCYPs.

| **S.No.** | **miRNA_Acc.** | **Target_Acc.** |
| --- | --- | --- |
| 1 | tae-miR1120c-5p | TaCYP-17 |
| 2 | tae-miR1122a | TaCYP-23 |
| 3 | tae-miR1137b-5p |  |
| 4 | tae-miR1128 | TaCYP-24 |
| 5 | tae-miR1137a |  |
| 6 | tae-miR1137b-5p |  |
| 7 | tae-miR1122a | TaCYP-26 |
| 8 | tae-miR9677a | TaCYP-35 |
| 9 | tae-miR1127a | TaCYP-36 |
| 10 | tae-miR1127a | TaCYP-41 |
| 11 | tae-miR1127b-3p |  |
| 12 | tae-miR1133 |  |
| 13 | tae-miR1120b-3p | TaCYP-43 |
| 14 | tae-miR1122c-3p |  |
| 15 | tae-miR1122c-3p |  |
| 16 | tae-miR1130a |  |
| 17 | tae-miR1130b-3p |  |
| 18 | tae-miR1130b-3p |  |
| 19 | tae-miR1130b-3p | TaCYP-6 |
| 20 | tae-miR1130a | TaCYP-61 |
| 21 | tae-miR1130a | TaCYP-62 |
| 22 | tae-miR1137a | TaCYP-64 |
| 23 | tae-miR1137b-5p |  |
| 24 | tae-miR1121 | TaCYP-65 |
| 25 | tae-miR1127a | TaCYP-67 |
| 26 | tae-miR1127b-3p |  |
| 27 | tae-miR1133 |  |
| 28 | tae-miR1130b-3p | TaCYP-68 |
| 29 | tae-miR1121 | TaCYP-7 |
| 30 | tae-miR1127b-3p |  |
| 31 | tae-miR1122b-3p | TaCYP-70 |
| 32 | tae-miR1122c-3p |  |
| 33 | tae-miR1127a |  |
| 34 | tae-miR1130b-3p |  |
| 35 | tae-miR1136 |  |
| 36 | tae-miR1130a | TaCYP-76 |
| 37 | tae-miR1130b-3p |  |
| 38 | tae-miR1120b-3p | TaCYP-81 |
| 39 | tae-miR1130a |  |
| 40 | tae-miR1130b-3p |  |
