# Supplementary figures and images for: Genome-wide characterization and identification of cyclophilin genes associated with leaf rust resistance in bread wheat (Triticum aestivum L.)
Source: Front Genet. 2022 Sep 30;13:972474. doi: 10.3389/fgene.2022.972474 (PMC9561851; doi:10.3389/fgene.2022.972474)

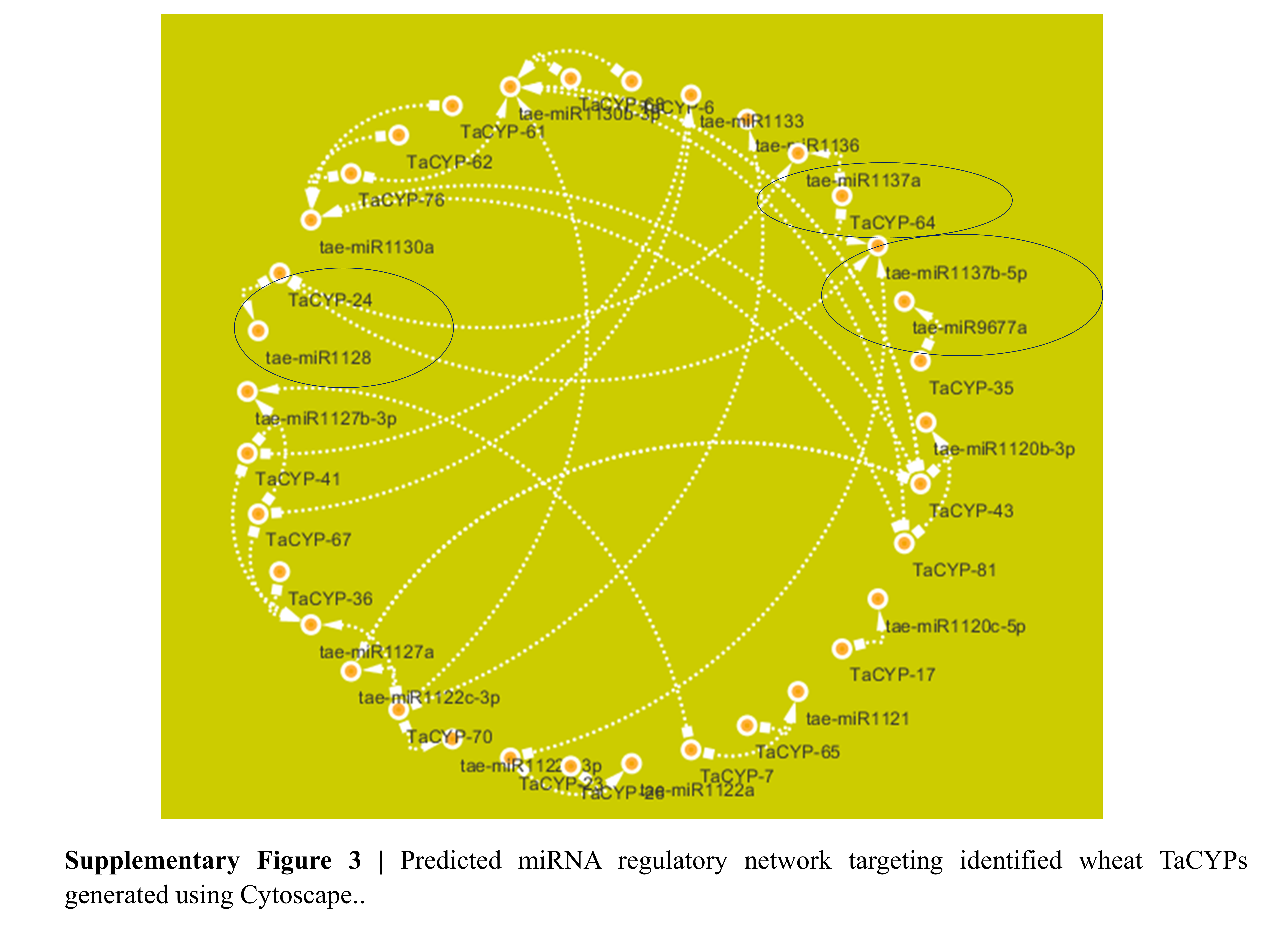

Supplement: Supplementary file 2 [file Image3.TIF]

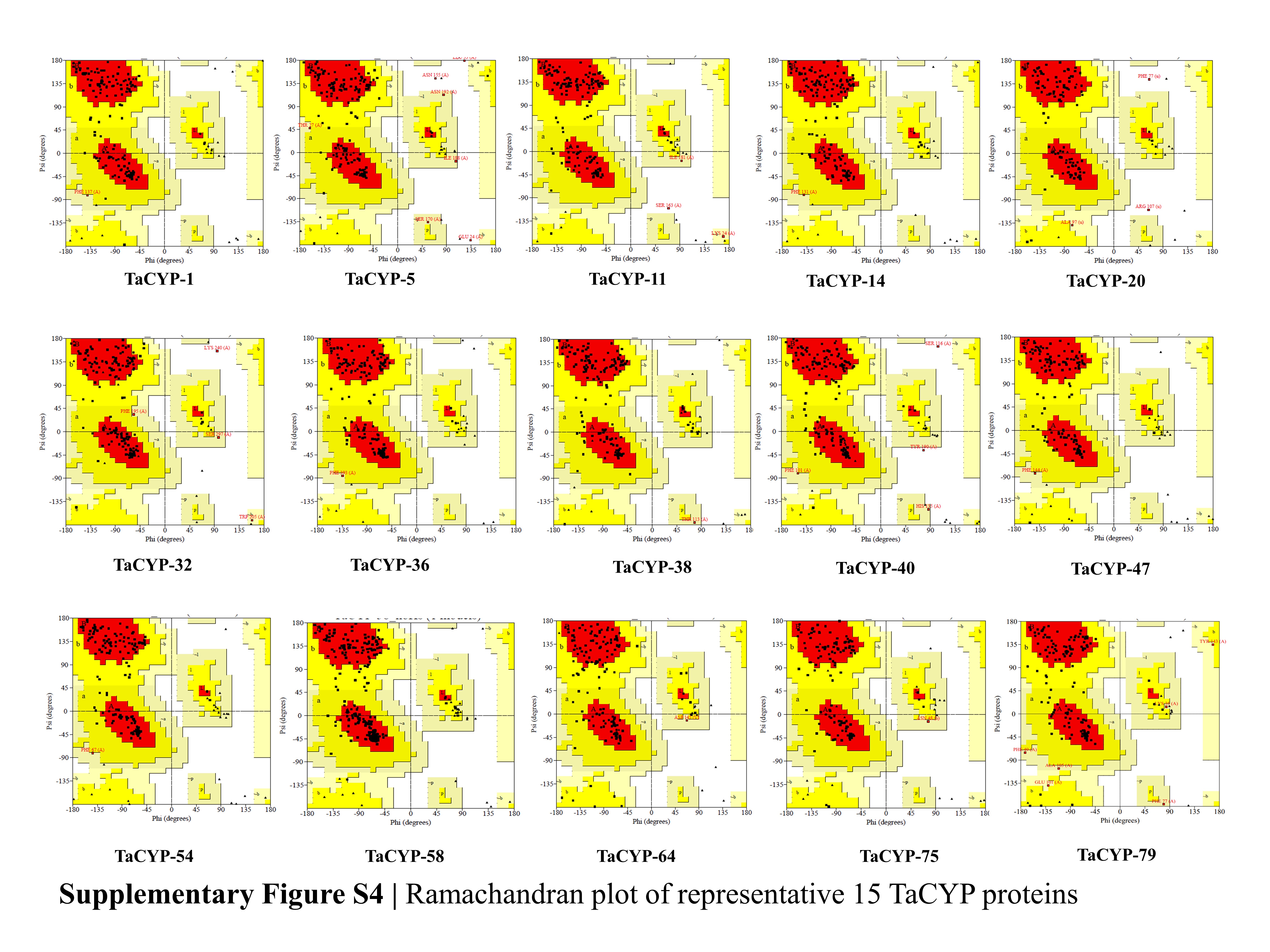

Supplement: Supplementary file 3 [file Image4.TIF]

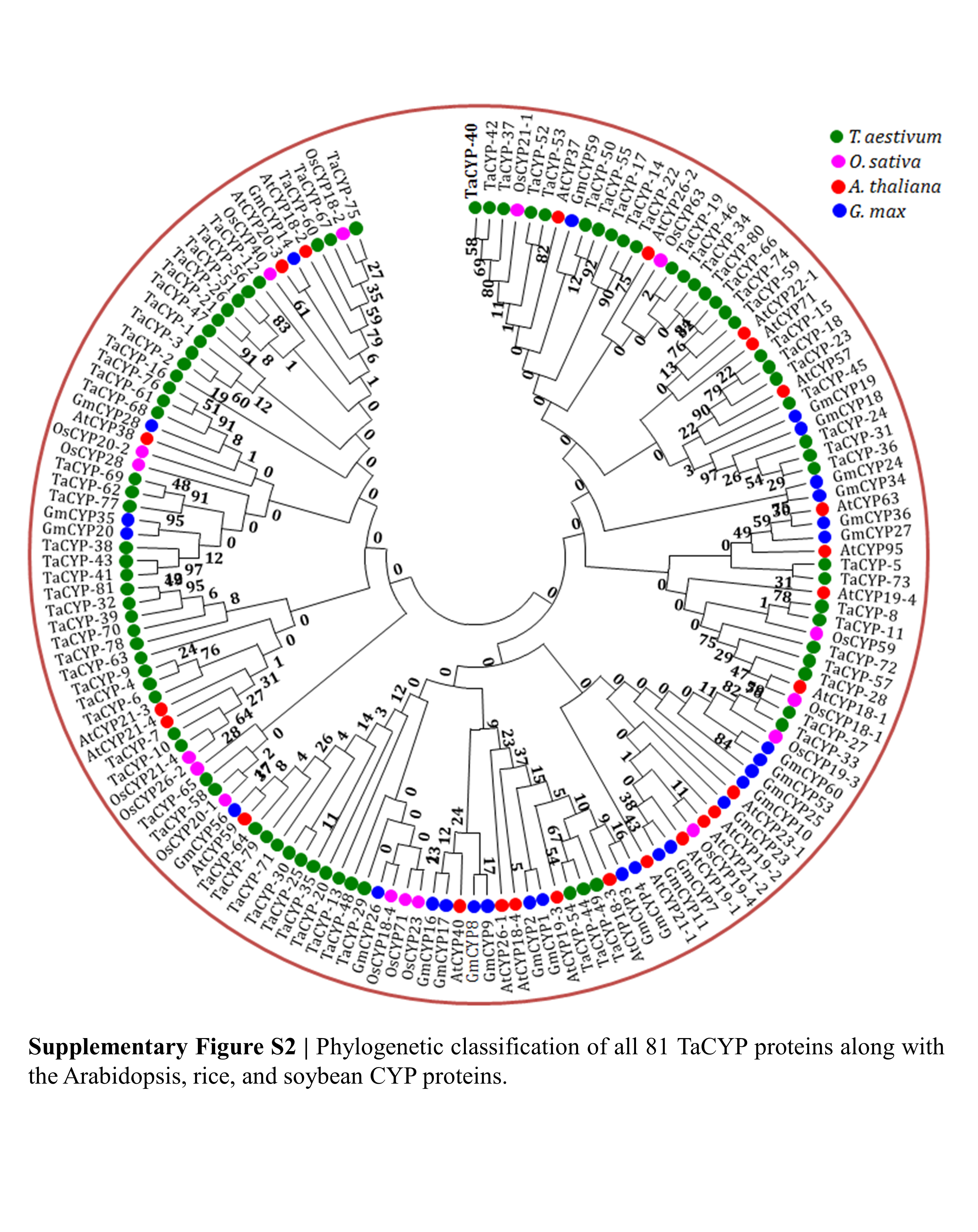

Supplement: Supplementary file 4 [file Image2.TIF]

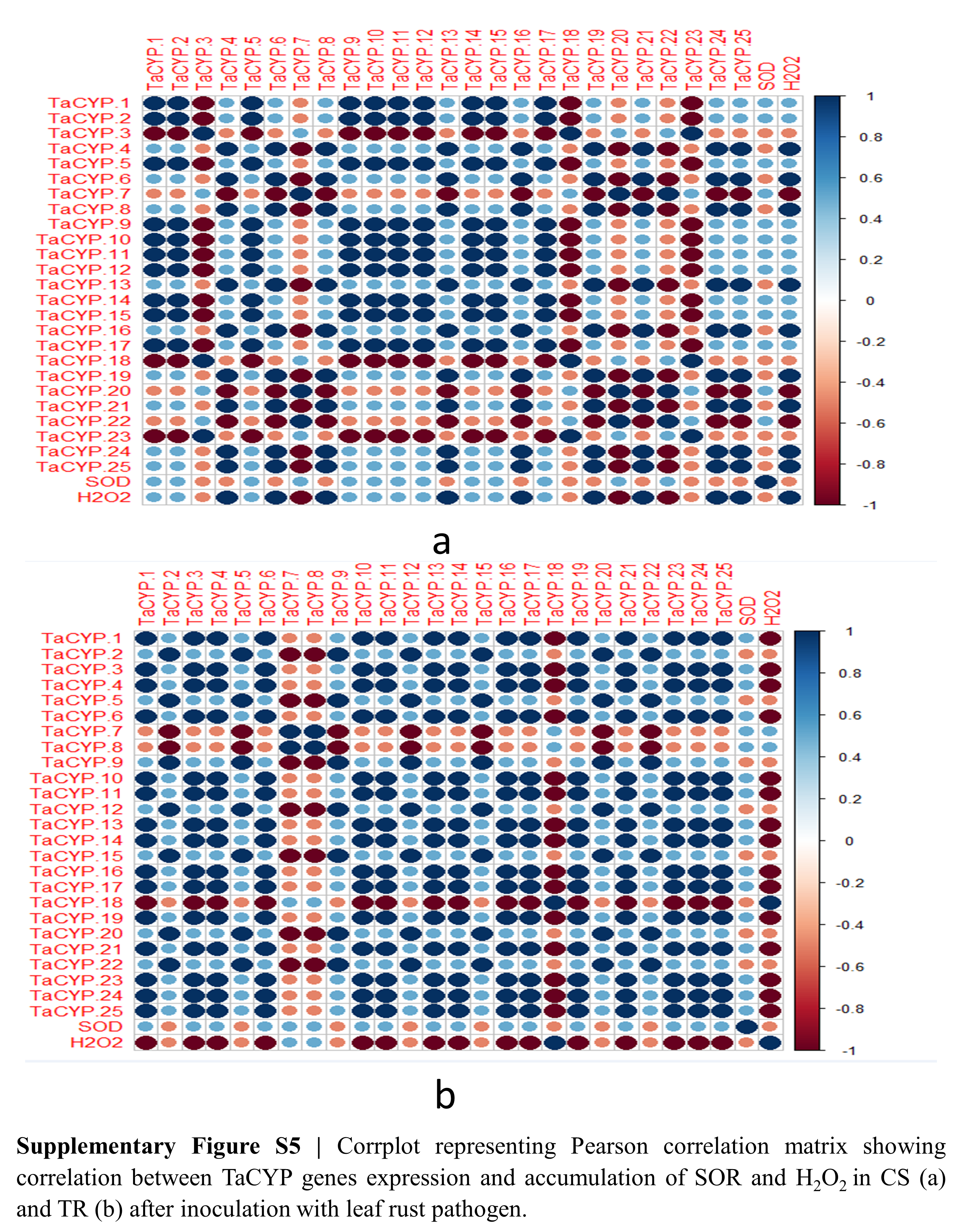

Supplement: Supplementary file 8 [file Image5.TIF]
