# Supplementary material for: Genome-wide characterization and identification of cyclophilin genes associated with leaf rust resistance in bread wheat (Triticum aestivum L.)
Source: Front Genet. 2022 Sep 30;13:972474. doi: 10.3389/fgene.2022.972474 (PMC9561851; doi:10.3389/fgene.2022.972474)
Supplement: Supplementary file 6 [file Table2.DOCX]

**Suppl. Table 2:** Details of 25 primers designed for qRT-PCR

| **Chromosome** | **Primer** | **Grouped *TaCYP* genes** | **bp** | **aa** | **Exons (Coding exons)** | **Stand** | **Sequence** | **Length** | **Tm** | **GC%** |
| --- | --- | --- | --- | --- | --- | --- | --- | --- | --- | --- |
| 1 | *XTaCYP-*1 | *TaCYP*1(1A) | 1146 | 245 | 7 (7) | Forward | CGAAGAAGAAGGTGGTCATTAG | 22 | 60 | 45.5 |
|  |  | *TaCYP*2(1B) | 1350 | 245 | 7(7) | Reverse | CCGTATGCCTCAGAGTAGAA | 20 | 60 | 50 |
|  |  | *TaCYP*3 (1D) | 1322 | 245 | 7(7) |  |  |  |  |  |
| 2 | *XTaCYP-*2 | *TaCYP*4 (1A) | 1640 | 459 | 13 (12) | Forward | CCAACCCAGTTCTTCTTCTATC | 22 | 60 | 45.5 |
|  |  | *TaCYP*6 (1B) | 2857 | 459 | 12 (12) | Reverse | CGAATCTTATCTCCAGCCTTAAT | 23 | 60 | 39.1 |
|  |  | *TaCYP*9 (1D) | 2863 | 424 | 12 (12) |  |  |  |  |  |
|  | *XTaCYP-*3 | *TaCYP*5 (2A) | 2404 | 643 | 14 (13) | Forward | GGTTCGGAGTCACAATCATAC | 21 | 60 | 47.6 |
|  |  | *TaCYP*8 (2B) | 2453 | 635 | 14 (13) | Reverse | TCCTCTACTCTTGCTCTTACTC | 22 | 60 | 45.5 |
|  |  | *TaCYP*11 (2D) | 2437 | 636 | 14 (13) |  |  |  |  |  |
|  | *XTaCYP-*4 | *TaCYP*7 (2B) | 1282 | 233 | 8 (7) | Forward | CTGGTGTTGTGGACAGATTTA | 21 | 60 | 42.9 |
|  |  | *TaCYP*10 (2D) | 1105 | 233 | 8 (7) | Reverse | TGGCTTTGGCTTTCAGTATC | 20 | 61 | 45 |
| 3 | *XTaCYP-*5 | *TaCYP*12 (3A) | 1148 | 295 | 2 (2) | Forward | CCCTACACCAAGATCCTAGTAA | 22 | 60 | 45.5 |
|  |  | *TaCYP*16 (3B) | 1181 | 291 | 2 (2) | Reverse | GTGGACGACAGGAAAGAATG | 20 | 60 | 50 |
|  |  | *TaCYP*19 (3D) | 1108 | 295 | 2 (2) |  |  |  |  |  |
|  | *XTaCYP-*6 | *TaCYP*14 (3A) | 1207 | 240 | 6 (6) | Forward | GCTCCTTCCACCGAATTATC | 20 | 60 | 50 |
|  |  | *TaCYP*17 (3B) | 723 | 240 | 6 (6) | Reverse | TAGTACACCAGGACCAACAT | 20 | 60 | 45 |
|  |  | *TaCYP*22 (3D) | 1160 | 240 | 6 (6) |  |  |  |  |  |
|  | *XTaCYP-*7 | *TaCYP*15 (3A) | 1977 | 495 | 11 (10) | Forward | GTCATCAAGTCCTTCCTCATC | 21 | 60 | 47.6 |
|  |  | *TaCYP*18 (3B) | 1868 | 500 | 11 (10) | Reverse | CAGCGGTCAAGGGTAATAAA | 20 | 60 | 45 |
|  |  | *TaCYP*23 (3D) | 1894 | 499 | 11 (10) |  |  |  |  |  |
| 4 | *XTaCYP-*8 | *TaCYP*24 (4A) | 2144 | 590 | 11 (11) | Forward | CTGAAGCAGGAGGATCTAATTC | 22 | 60 | 45.5 |
|  |  | *TaCYP*31 (4B) | 2216 | 590 | 11 (11) | Reverse | CTCCTGGATTGCCTCATAAC | 20 | 60 | 50 |
|  |  | *TaCYP*36 (4D) | 2217 | 591 | 11 (11) |  |  |  |  |  |
|  | *XTaCYP-*9 | *TaCYP*25 (4A) | 1437 | 478 | 1 (1) | Forward | CGTGGAATACCTCGATGATAAA | 22 | 60 | 40.9 |
|  |  | *TaCYP*30 (4B) | 1401 | 466 | 2 (2) | Reverse | TCAGGGTCATACAACACATAAG | 22 | 60 | 40.9 |
|  |  | *TaCYP*35 (4D) | 1964 | 481 | 2 (1) |  |  |  |  |  |
|  | *XTaCYP-*10 | *TaCYP*26 (4A) | 1112 | 237 | 8 (8) | Forward | CGATGTCATGGGAGGTAGAA | 20 | 61 | 50 |
|  |  | *TaCYP*34 (4D) | 824 | 231 | 8 (8) | Reverse | GCATCACCAAGGAGGATAGA | 20 | 61 | 50 |
| 5 | *XTaCYP-*11 | *TaCYP*37 (5A) | 1159 | 216 | 7 (7) | Forward | ATCTGTCCTCCCTCCTATTC | 20 | 60 | 50 |
|  |  | *TaCYP*40 (5B) | 1013 | 216 | 7 (7) | Reverse | CAGCTATTCTGCTCGTATTACA | 22 | 60 | 40.9 |
|  |  | *TaCYP*42 (5D) | 1084 | 216 | 7 (7) |  |  |  |  |  |
|  | *XTaCYP-*12 | *TaCYP*38 (5A) | 1023 | 198 | 7 (7) | Forward | ATTCATCGCTTCTGCTACTG | 20 | 60 | 45 |
|  |  | *TaCYP*41 (5B) | 997 | 198 | 7 (7) | Reverse | GCAGAATAAGCCACAGAAATAAG | 23 | 60 | 39.1 |
|  |  | *TaCYP*43 (5D) | 981 | 198 | 7 (7) |  |  |  |  |  |
| 6 | *XTaCYP-*13 | *TaCYP*44 (6A) | 973 | 171 | 1 (1) | Forward | GTCGTGTCGTCGATCTAAATAA | 22 | 60 | 40.9 |
|  |  | *TaCYP*49 (6B) | 903 | 171 | 1 (1) | Reverse | GCAGACACAGGAAGATAACC | 20 | 60 | 50 |
|  |  | *TaCYP*54 (6D) | 969 | 171 | 1 (1) |  |  |  |  |  |
|  | *XTaCYP-*14 | *TaCYP*45 (6A) | 3069 | 808 | 15 (13) | Forward | CTGAGGATGCCAGAAGAAAG | 20 | 60 | 50 |
|  |  | *TaCYP*50 (6B) | 3052 | 849 | 15 (14) | Reverse | TACTCCAGCTTACACCAAATG | 21 | 60 | 42.9 |
|  |  | *TaCYP*55 (6D) | 3035 | 849 | 15 (14) |  |  |  |  |  |
|  | *XTaCYP-*15 | *TaCYP*46 (6A) | 1690 | 406 | 10 (9) | Forward | TGCCTGTAACTTTCCGATTAG | 21 | 60 | 42.9 |
|  |  | *TaCYP*51 (6B) | 1680 | 408 | 10 (9) | Reverse | AGATACCAGAAAGGCAGAAAC | 21 | 60 | 42.9 |
|  |  | *TaCYP*56 (6D) | 1680 | 408 | 10 (9) |  |  |  |  |  |
| 7 | *XTaCYP-*16 | *TaCYP*57 (7A) | 1002 | 160 | 2 (2) | Forward | CCGTCCGGTTCTGTAATATC | 20 | 60 | 50 |
|  |  | *TaCYP*72 (7D) | 803 | 160 | 3 (2) | Reverse | TAAGTAGCAAGCAGCAAGAG | 20 | 60 | 45 |
|  | *XTaCYP-*17 | *TaCYP*58 (7A) | 1747 | 379 | 9 (8) | Forward | GCTGGATGTGTATCTCCTATTC | 22 | 60 | 45.5 |
|  |  | *TaCYP*65 (7B) | 1748 | 380 | 9 (8) | Reverse | AGCCATGTAGCACTCTATCTA | 21 | 60 | 42.9 |
|  |  | *TaCYP*73 (7D) | 1777 | 375 | 9 (8) |  |  |  |  |  |
|  | *XTaCYP-*18 | *TaCYP*59 (7A) | 2738 | 648 | 15 (13) | Forward | GCTGTTAGTGACCGGAATAC | 20 | 60 | 50 |
|  |  | *TaCYP*66 (7B) | 1947 | 648 | 13 (13) | Reverse | CATCCTGGGCATGGTTATAC | 20 | 60 | 50 |
|  |  | *TaCYP*74 (7D) | 2435 | 648 | 15 (13) |  |  |  |  |  |
|  | *XTaCYP-*19 | *TaCYP*60 (7A) | 798 | 164 | 6 (6) | Forward | GGGCTGGGATTCTATCAATG | 20 | 60 | 50 |
|  |  | *TaCYP*67 (7B) | 907 | 164 | 6 (6) | Reverse | CCAAGGCGCTTAACAATTTC | 20 | 60 | 45 |
|  |  | *TaCYP*75 (7D) | 927 | 154 | 7 (6) |  |  |  |  |  |
|  | *XTaCYP-*20 | *TaCYP*61 (7A) | 1706 | 423 | 7 (7) | Forward | CTCAAGAGCGCTGACTAATG | 20 | 60 | 50 |
|  |  | *TaCYP*68 (7B) | 1574 | 423 | 7 (7) | Reverse | CTACCGCATTCCTGTTTCTATC | 22 | 61 | 45.5 |
|  |  | *TaCYP*76 (7D) | 1857 | 431 | 7 (7) |  |  |  |  |  |
|  | *XTaCYP-*21 | *TaCYP*62 (7A) | 984 | 213 | 7 (7) | Forward | GAATGGACGTGGTCTACAAG | 20 | 60 | 50 |
|  |  | *TaCYP*69 (7B) | 1016 | 213 | 7 (7) | Reverse | CCCGACAATAACGGTTCTAC | 20 | 60 | 50 |
|  |  | *TaCYP*77 (7D) | 1109 | 213 | 7 (7) |  |  |  |  |  |
|  | *XTaCYP-*22 | *TaCYP*63 (7A) | 1503 | 406 | 3 (3) | Forward | CCTGCACTACTTCTGCATATC | 21 | 60 | 47.6 |
|  |  | *TaCYP*70 (7B) | 1521 | 409 | 3 (3) | Reverse | CGCTTTAGGATCAACAGTCTC | 21 | 60 | 47.6 |
|  |  | *TaCYP*78 (7D) | 1450 | 413 | 3 (3) |  |  |  |  |  |
|  | *XTaCYP-*23 | *TaCYP*64 (7A) | 2255 | 559 | 14 (14) | Forward | GATCACATAGCCCGAGATTG | 20 | 60 | 50 |
|  |  | *TaCYP*71 (7B) | 2189 | 559 | 14 (14) | Reverse | CAGTATTCCGATGAGCAGTATC | 22 | 60 | 45.5 |
|  |  | *TaCYP*79 (7D) | 2204 | 559 | 14 (14) |  |  |  |  |  |
| UN | *XTaCYP-*24 | *TaCYP*80 | 1189 | 231 | 8 (8) | Forward | GTATTTGGGAGGGTGACTAAG | 21 | 60 | 47.6 |
|  |  |  |  |  |  | Reverse | GCTTCACAGCTCTCTACATC | 20 | 60 | 50 |
| UN | *XTaCYP-*25 | *TaCYP*81 | 1269 | 325 | 5 (5) | Forward | CCGGTGTATATCAAGGTGAAG | 21 | 60 | 47.6 |
|  |  |  |  |  |  | Reverse | CTCTTCCAAACCTCCTCTATTT | 22 | 60 | 40.9 |
