# Supplementary material for: Genome-wide characterization and identification of cyclophilin genes associated with leaf rust resistance in bread wheat (Triticum aestivum L.)
Source: Front Genet. 2022 Sep 30;13:972474. doi: 10.3389/fgene.2022.972474 (PMC9561851; doi:10.3389/fgene.2022.972474)
Supplement: Supplementary file 7 [file Table3.DOCX]

**Suppl. Table 3**: Different statistical values of calculated Ramachandran plots for representative 15 TaCYPs protein models.

| **Protein** | **Residues in most favored regions**  **(%)** | **Residues in additional allowed regions (%)** | **Residues in generously allowed regions (%)** | **Residues in disallowed regions**  **(%)** |
| --- | --- | --- | --- | --- |
| TaCYP-1 | 87.5 | 11.8 | 0.7 | 0.0 |
| TaCYP-5 | 83.2 | 12.3 | 2.6 | 1.9 |
| TaCYP-11 | 85.9 | 12.1 | 1.3 | 0.7 |
| TaCYP-14 | 86.9 | 12.4 | 0.7 | 0.0 |
| TaCYP-20 | 86.3 | 11.1 | 0.9 | 1.7 |
| TaCYP-32 | 84.9 | 12.3 | 2.1 | 0.7 |
| TaCYP-36 | 86.3 | 13.0 | 0.7 | 0.0 |
| TaCYP-38 | 81.0 | 18.3 | 0.7 | 0.0 |
| TaCYP-40 | 82.7 | 14.3 | 1.5 | 1.5 |
| TaCYP-47 | 86.6 | 12.7 | 0.7 | 0.0 |
| TaCYP-54 | 88.2 | 11.0 | 0.7 | 0.0 |
| TaCYP-58 | 90.2 | 9.8 | 0.0 | 0.0 |
| TaCYP-64 | 77.9 | 21.5 | 0.7 | 0.0 |
| TaCYP-75 | 86.9 | 12.3 | 0.8 | 0.0 |
| TaCYP-79 | 77.7 | 21.6 | 0.7 | 0.0 |
